# Synthesis and Biological Evaluation of New Substituted Hantzsch Thiazole Derivatives from Environmentally Benign One-Pot Synthesis Using Silica Supported Tungstosilisic Acid as Reusable Catalyst

**DOI:** 10.3390/molecules22050757

**Published:** 2017-05-07

**Authors:** Houria Bouherrou, Aicha Saidoun, Ahmed Abderrahmani, Lamia Abdellaziz, Yahia Rachedi, Françoise Dumas, Albert Demenceau

**Affiliations:** 1 Laboratory of Applied Organic Chemistry, Faculty of Chemistry, University of Science and Technology Houari Boumediene, BP 32, El Alia, 16111 Bab Ezzouar, Algiers, Algeria; houriab87@yahoo.fr (H.B.); aicha.saidoun@hotmail.com (A.S.); 2 Laboratory of Cellular and Molecular Biology, Faculty of Biological Sciences, USTHB, BP 32, El Alia, 16111 Bab Ezzouar, Algiers, Algeria; abderrahmaniahmed1@gmail.com (A.A.); abdelaziz.lm@hotmail.com (L.A.); 3 Laboratoire BioCIS, UMR CNRS8076, Chimie des Substances Naturelles, IPSIT and LabEx LERMIT, CNRS, Université Paris-Saclay, Université Paris-Sud, Faculté de Pharmacie, 5, rue Jean-Baptiste Clément, 92296 Châtenay-Malabry CEDEX, France; fran_dumas@yahoo.com; 4 Laboratoire de chimie macromoléculaire et catalyse organique, Institut de Chimie (B6a), Université de Liège, Quartier Agora, Allée du 6 Août, 4000 Liège (Sart-Tilman), Belgique; albertdemanceau@gmail.com

**Keywords:** thiazoles, multi-component reaction, Hantzsch condensation, heterogeneous catalysis, antioxidant, antibacterial

## Abstract

An efficient and green method has been developed for the synthesis of new substituted Hantzsch thiazole derivatives in 79%–90% yield, via the one-pot multi-component procedure, by the reaction of 3-(bromoacetyl)-4-hydroxy-6-methyl-2*H*-pyran-2-one, thiourea and substituted benzaldehydes in the presence of silica supported tungstosilisic acid, as a reusable catalyst, under conventional heating or under ultrasonic irradiation. The catalyst is recoverable by a simple filtration and can be reused in the subsequent reactions. Most of the thiazoles exhibited significant antibacterial activity compared toamoxicillin and ciprofloxacin as positive controls. In addition, the new compounds showed moderate to good antioxidant (DPPH) radical scavenging activity.

## 1. Introduction

Molecular entities bearing thiazole ring system(s) form an important class of natural and synthetic compounds because of their physicochemical properties [1,2,3,4].Furthermore, they exhibit a wide range of biological activities such as cardiotonic [5], antifungal [6,7], analgesic [8], anticonvulsant [9], antituberculosis [10], antiviral [11], anti-inflammatory [12], anti-HIV [13,14], and anticancer activities [15,16].

It is known that thiazoles can be synthesized from α-bromoketone and a thiourea via Hantzsch thiazole synthesis in high yields [17]. A literature survey revealed that there are numerous routes reported for the synthesis of substituted thiazoles according to Hantzsch thiazole synthesis [18,19].

Due to the importance of these heterocycles in medicinal and material chemistry, the development of new routes—which leads to these heterocycles in higher yields, shorter reaction time, milder or greener conditions—has received considerable attention in organic synthesis [20,21,22,23,24,25,26,27]. When designing organic synthesis, chemists have to face the ecocompatibility concern of their synthesis plan. Multi-component reactions (MCRs) are a special type of synthetically useful organic reaction, in which, three or more different starting materials react, to generate a single product in a one-pot procedure [28,29,30,31,32]. Thus, MCRs also represent a possible instrument to perform near ideal synthesis, because they possess one of the aforementioned qualities, namely the possibility of building up complex molecules with maximum simplicity and brevity [33,34]. On the other hand, the use of catalysis in organic synthesis has received considerable attention owing to its important advantages: it helps to reduce energy consumptionand increases control of selectivity [35,36,37].In particular, heterogeneous catalysis appears as the solution of choice since many groups have clearly shown that the molecular structure and/or texture of catalytic materials could directly affect the selectivity of the reactions involved [38,39]. Heteropolyacids (HPAs) are widely used as heterogeneous catalysts and offer many advantages, especially polyoxometalate compounds such as tungstosilisic acid H_4_SiW_12_O_40_ (SiW) [36,37].

**Scheme 1:**
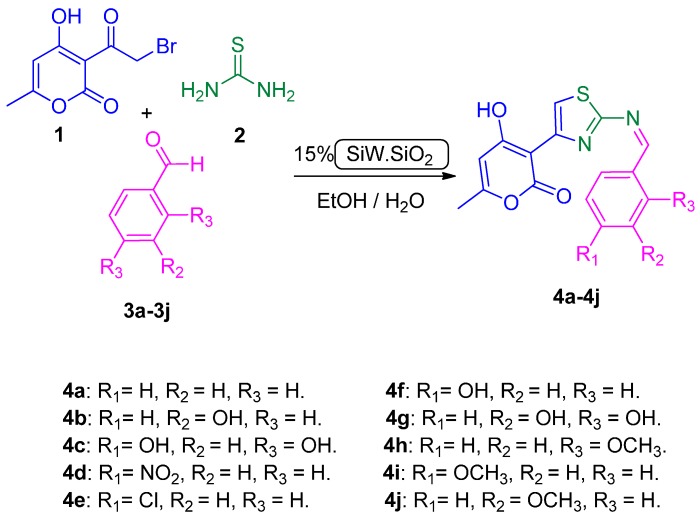
SiW.SiO_2_ catalyzed synthesis of new Hantzsch thiazole derivatives **4a**–**4j**.

Despite its unusual activation mode, ultrasonic irradiation **(**US**)** has been increasingly used in organic synthesis in the last three decades [40,41]. A large number of organic reactions can be carried out in higher yields, shorter reaction time and milder conditions under ultrasound irradiation [42,43]. In comparison to conventional methods, the US method is more convenient and easily controlled [44,45,46]. Owing to the above facts and in continuation of our research work on the development of MCRs for the synthesis of novel heterocyclic compounds, in this article, we report on the synthesis of functionalized thiazoles, in good yields, using SiW.SiO_2_ as a reusable solid support catalyst (Scheme 1) and their biological evaluation.

## 2. Results and Discussion

### 2.1. Chemistry

The required α-haloketone (**1**), 3-(bromoacetyl)-4-hydroxy-6-methyl-2*H*-pyran-2-one, is not commercially available. It could be easily obtained via the selective α-monobromination of dehydroacetic acid (DHAA) (**5**) in 70% yield according to a known procedure [47].

In that way, we decided to study the reactivity of (**1**) with several binucleophilic amines and initiate our studies with the reaction of thiourea (**2**) to afford the corresponding thiazoles. The thiazoles were assembled according to a slightly modified version of the Hantzsch thiazoles synthesis [48,49]. Herein, the three-component one-pot condensation of an equimolar amount of (**1**), (**2**) and substituted benzaldehydes (**3a**–**3j**), using SiW.SiO_2_ as a catalyst under conventional heating and under ultrasonic irradiation, yielded the corresponding thiazole derivatives (**4a**–**4j**) in good to excellent yield. In order to enhance the process, the reaction condition was optimized for the transformation of (**1**), (**2**) and benzaldehyde (**3a**) under different conditions of temperature, solvents and amount of catalyst. The observed results are summarized in Table 1.

Initially, we investigated the effect of various solvents in this model reaction. The above 3-(bromoacetyl)-4-hydroxy-6-methyl-2*H*-pyran-2-one (**1**), thiourea (**2**) and benzaldhyde (**3a**) were stirred in various solvents, such as water, ethanol, methanol, 1-butanol and 2-propanol at their respective reflux condition (Entries 2, 4, 6–8, Table 1). The reaction smoothly proceeded in 1-butanol, 2-propanol and water under reflux condition but not at ambient temperature (25 °C). These conditions remained unsuccessful for obtaining acceptable yields (Entries 1, 3, 5, Table 1), whereas the use of a mixture of ethanol/water (50/50 *v*/*v*) at reflux allowed the desired product in higher yield, typically from 50% to 87% (entries 10–16, Table 1). Therefore, the 1 to 1 mixture of ethanol/water represents the suitable, cheap, safe and environmentally benign solvent for the present investigation.

We further examined the amount of SiW.SiO_2_ required for this reaction under the above conditions. The effect of catalyst amount on the multi-component reaction was investigated by varying the catalyst amount (5%, 10%, 15%, 18% and 20%). It was found that when increasing the amount of the SiW.SiO_2_ from 5% to 15%, the yield also increased (Table 1). A further increase of the catalyst loading (above 15%) does not affect the yield. Therefore, 15% SiW.SiO_2_ in the mixture of ethanol/water is sufficient to push this reaction forward. Next, we investigated the reusability of SiW.SiO_2_. At the end of the reaction, the catalyst could be recovered by a simple filtration. The recycled catalyst could be washed well with acetone and subjected to a second run of the reaction process. To ensure that the catalyst did not dissolve in acetone, it was weighted before using it then after filtration and reuse for the next reaction. The results show that this catalyst is not soluble in acetone. The catalyst remained active and exhibited no substantial loss of activity over up to three reaction cycles. The yield gradually decreased (72%) for the fourth run. This may be due to loss of SiW on the SiO_2_ surface area.

Encouraged by the results obtained for the synthesis of **4a**, we extended this methodology to various substituted benzaldehydes (**3b**–**3j**), to prepare the corresponding thiazole derivatives (**4b**–**4j**) by two methods: under conventional heating (method A) and under ultrasonic irradiation (method B). By using ultrasonic activation assisted synthesis, at room temperature, the reaction time could be decreased compared to the previously reported conventional method as worked out for some analogs (2h compared to 3.5h), without affecting the yield. The scope and generality of this process are illustrated with respect to various substituted benzaldehydes as shown in Table 2.

**Scheme 2:**
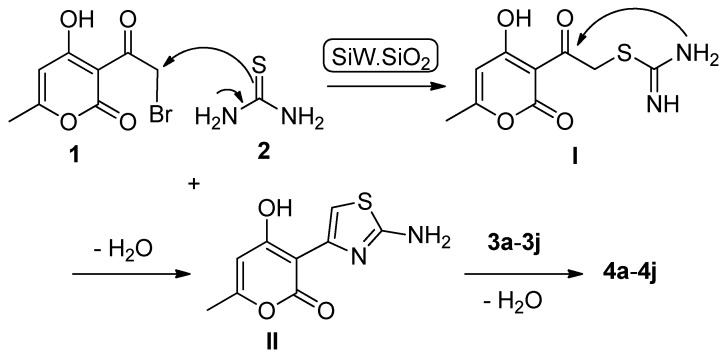
A plausible mechanism for the formation of Hantzsch thiazole derivatives.

In order to obtain more insight into the mechanism of the functionalized thiazoles formation, the stepwise reaction was studied. Thus, the intermediate (**II**) had been prepared by cyclocondensation of (**1**) with (**2**) in a mixture of ethanol and water (50/50, *v*/*v*) under ultrasonic irradiation. The reaction of an equimolar amount of (**II**) with (**3a**) in the same conditions gave (**4a**) with a yield of 60%. The characterisation of (**II**) had been reported in our previous work [50]. Our experiments showed that the target thiazole derivatives can be obtained “in one flask” by initially conducting cyclocondensation of the α-haloketone (**1**) with thiourea (**2**) followed by condensation of the intermediaite (**II**)with substituted benzaldehydes (**3a**–**3j**) whichsupport the plausible mechanism as depicted in Scheme 2.

All the new target compounds were completely characterized by using infrared (IR), ^1^H-NMR, ^13^C-NMR and mass spectroscopy (see supplementary). The spectroscopic data of the new compounds are given in the experimental section and are fully consistent with the proposed structures. IR spectra of **4a**–**4j** had strong N=C absorptions at about 1669 cm^−1^ and displayed absorptions at about 1617–1546 cm^−1^ and 1582 cm^−1^ which were assigned to C=O and C=C functionalities respectively. This study confirmed the conservation of 2-pyroneby the presence of large elongation and intense bands in the absorption range at 1718–1693 cm^−1^. The ^1^H-NMR spectra of the compounds **4a**–**4j** exhibited broad signals at 6.02–6.92 ppm, which were assigned to the C–H proton of the thiazole ring. ^13^C-NMR of compounds **4a**–**4j** showed peaks at about 111.98–115.10 and 164.33–167.46 for C-S (thiazole) and C=N (amide), respectively.

### 2.2. Biological Evaluation

#### 2.2.1. Antioxidant Activity

The 2,2-diphenyl-1-picrylhydrazil (DPPH) is a stable free radical that has been widely accepted as a tool for estimating the free radical scavenging activities of antioxidants [51]. The lower IC_50_ value indicates a stronger ability of the compound to act as a DPPH scavenger, while the higher IC_50_ value indicates a lower scavenging activity of the scavengers. This is because more scavengers were required to achieve a 50% scavenging reaction. In reaction with a hydrogen donor, the purple color of DPPH fades or disappears, due to its conversion to 2,2-diphenyl-1-picryl hydrazine, resulting in an absorbance decrease. The greater the decrease in absorption, in the presence of scavengers in the fractions, the more effective is its antioxidant activity.

**Figure 1:**
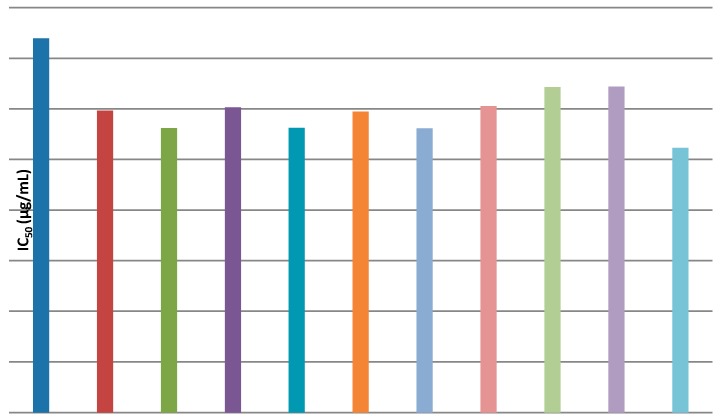
The antioxidant activity of compounds (**4a**–**4j**) using DPPH assay (IC_50_= μg/mL), compared with reference standard butylhydroxytoluene (BHT) activity.

In the current study, the antioxidant activity of compounds (**4a**–**4j**) was expressed as IC_50_ with a low IC_50_ value, indicating that the synthesis compounds act as an effective DPPH scavenger (Figure 1). IC_50_ values were between 11.23 and 16.96 μg/mL for the compounds (**4a**–**4j**), while 10.46 μg/mL for butylhydroxytoluene (BHT, positive control). The newly synthesized thiazoles display significant antioxidant capacitywith an insignificant variation, which indicates that the thiazole ring enhances the antioxidant activity of thiazoles, not the benzene ring where the variation is. However, compounds **4b**, **4c** and **4g** with both electrons donating and electron withdrawing groups in para position exhibited a promising antioxidant (DPPH) radical scavenging activity compared to BHT.

#### 2.2.2. In Vitro Antibacterial Activity

**Figure 2:**
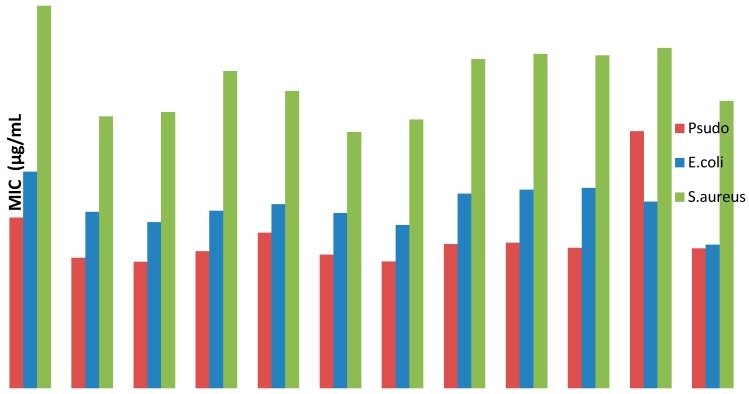
The antibacterial activity of compounds (**4a**–**4j**) compared to Ciprofloxacin and Amoxicillin (MIC = μg/mL).

The in vitro antibacterial activity of compounds (**4a**–**4j**), expressed as an MIC value, was assessed by the serial dilution methods against *Escherichia coli*, *Staphylococcus aureus*, *Pseudomonas aeruginosa* and two standard antibiotics (amoxicillin and ciprofloxacin) (Figure 2). The MICs of the synthesis compounds were within concentration ranges of 50–153 μg/mL. The entire synthesized compounds and standard antibiotics had higher antibacterial activity against bacteria strains and are most active against Gram-negative bacteria than the Gram-positive bacteria. In general, all compounds are, approximately, behaving as the reference compounds with same activities. This potent antibacterial activity exhibited may be due to the thiazole ring. However, we observed that both electron donating as well as electron withdrawing groups were found to increase the antibacterial properties. Compounds **4b**, **4c**, **4f** and **4g** displayed moderate antibacterial activity whereas the remaining compounds showed less activity.

## 3. Experimental Section

### 3.1. Materials and Methods

All research chemicals and solvents were purchased from Sigma-Aldrich (mainly Sigma-Aldrich, Saint-Quentin-Fallavier, France) and were used as such for the reactions. The progress of all the reactions was monitored by thin-layer chromatography (TLC) using glass plates precoated with silica gel-60 F254 to a thickness of 0.5 mm. The melting points were taken in an open capillary tube using Electrothermal melting point apparatus (Electrotermal, Rochford, Great Britain). The values are reported in °C and are uncorrected. IR spectra were recorded as neat solid or liquid on a Fourier Transform Bruker Vector 22 spectrometer (Villebon-sur-Yvette, France). NMR spectra were recorded with a Bruker Avance 300 spectrometer [300 MHz (^1^H) and 75 MHz (^13^C)] (Bruker Biospin GmbH, Rheinstetten, Germany). Chemical shifts are expressed in parts per million (ppm) downfield from Tetramethylsilane (TMS). Data are reported as follows: chemical shift [multiplicity (s: singlet, d: doublet, dd: double doublet, ddd: double double doublet, dm: double multiplet, dt: double triplet, t: triplet, td triple doublet, tm, triple multiplet, tt: triple triplet, q: quartet, quint: quintuplet, m: multiplet, br: broad), coupling constants (*J*) in Hertz, integration]. The numbers of attached proton(s) in the ^13^C-NMR spectra were elucidated by use of JMOD experiments (DEPT 135) and are described as (CH_3_) methyl; (CH_2_) methylene; (CH) methine; (C) quaternary carbon atoms. Shifts of ^1^H- and ^13^C-NMR spectra were calibrated against the solvent residual isotopic peak as internal reference. Reference peaks for the NMR spectra in (CD_3_S)_2_O: 2.50 (^1^H) and 39.51 (^13^C) ppm. Mass spectra were recorded on a Waters Micromass LCT Premier Q-TOF Mass spectrometer (Waters, Guyancourt, France) coupled with a Waters Alliance HPLC. Synthesis under ultrasonic irradiation was carried out with 3510E-MT Bransonic ultrasonic apparatus (Bransonic, Danbury, CT, USA).

### 3.2. General Procedure for the Preparation of Compounds *(**4a**–**4j**)*

A mixture of 3-(bromoacetyl)-4-hydroxy-6-methyl-2*H*-pyran-2-one ) ((**1**) (1 mmol), thiourea (**2**) (1 mmol), benzaldehyde (**3a**–**3j**) (1 mmol) and SiW.SiO_2_(15%) [36] was refluxed in ethanol/water 1/1 (5 mL) with stirring for 2 h to 3.5 h at 65 °C or under ultrasonic activation for 1.5 h to 2 h at room temperature (Table 2).The obtained solid was filtered off and washed with ethanol; the remaining solid was dissolved in acetone and SiW.SiO_2_ was removed by filtration. The filtrated solution was evaporated under vacuum and the resulting product oven-dried (60 °C).

### 3.3. Characterization Data of Synthesized Compounds

*4-Hydroxy-6-methyl-3-(2-[-phenylmethylidene]amino-1,3-thiazol-4-yl)-2H-pyran-2-one* (**4a**). Yield 90%; Yellow solid; m.p. 220 °C; IR neat (cm^−1^): 3050–3500, 2957, 2165, 1683, 1616, 1494, 1450, 1358, 1314, 1249, 1172, 1031, 997, 866, 827, 719. ^1^H-NMR (300 MHz, (CD_3_S)_2_O), δ ppm: 2.21 (s, 3H, CH_3_), 5.52 (s, 1H, H-pyrone), 6.05 (s, 1H, H-thiazole), 7.25–7.72 (m, 5H, ArH), 7.93 (s, 1H, N=C-H), 9.16 (s, 1H, OH-pyrone); ^13^C-NMR (75 MHz, (CD_3_S)_2_O), δ ppm: 19.68 (H_3_C-pyrone), 90.29 (C-3), 99.50 (C-5), 123.05 (C-S thiazole), 127.84, 128.20, 128.52, 128.63, 129.52, 130.77 (ArC and ArCH), 132.91 (C-N at thiazole), 138.61 (C-6), 161.59 (C=N at thiazole), 164.54 (C-2), 167.35 (C=N), 169.25 (C-4); HRMS (ESI) *m*/*z* calc for C_16_H_13_N_2_O_3_S 313.0647, found 313.0645 [M + H]^+^.

*4-Hydroxy-3-(2-[-(3-hydroxyphenyl)methylidene]amino-1,3-thiazol-4-yl)-6-methyl-2H-pyran-2-one* (**4b**). Yield 88%. Red solid; m.p. 128 °C; IR neat (cm^−1^): 3050–3500, 2919, 1934, 1681, 1617, 1491, 1450, 1360, 1310, 1210, 1170, 1036, 997, 873, 824, 722. ^1^H-NMR (300 MHz, (CD_3_S)_2_O), δ ppm: 2.21 (s, 3H, CH_3_), 5.47 (s, 1H, H-pyrone), 6.05 (s, 1H, H-thiazole), 6.70 (d, *J* = 7.90 Hz, 1H, ArH), 7.10 (d, *J* = 7.78 Hz, 1H, ArH), 7.25 (s, 1H, ArH), 7.36 (s, 1H, N=C-H), 7.44 (t, *J_1_* = 7.90 Hz, *J_2_* = 7.78 Hz, 1H, ArH), 9.15 (s, 1H, ArOH), 9.91 (s,1H, OH-pyrone); ^13^C-NMR (75 MHz, (CD_3_S)_2_O) δ ppm: 19.80 (CH_3_-pyrone), 90.69 (C-3), 99.94 (C-5), 114.95(C-S at thiazole), 118.76, 121.53, 122.26, 123.60, 128.59 (ArC and ArCH), 129.94 (C-N at thiazole), 130.30 (ArC-OH), 140.53 (C-2), 157.69 (C=N thiazole), 164.46 (C-6), 167.46 (C=N), 169.66 (C-4); HRMS (ESI) *m*/*z* calc for C_16_H_13_N_2_O_4_S 329.0596, found 329.0597 [M + H]^+^.

*3-(2-[(2,4-Dihydroxyphenyl)methylidene]amino-1,3-thiazol-4-yl)-4-hydroxy-6-methyl-2H-pyran-2-one* (**4c**). Yield 82%. Brown solid; m.p. 237 °C; IR neat (cm^−1^): 3050–3500, 2956, 1718, 1681, 1613, 1503, 1450, 1358, 1284, 1209, 1167, 1036, 996, 862, 831, 716. ^1^H-NMR (300 MHz, (CD_3_S)_2_O), δ ppm: 2.20 (s, 3H, CH_3_), 5.43 (s, 1H, H-pyrone), 6.04 (s, 1H, H-thiazole), 6.44 (s, 1H, ArH), 6.65 (d, *J* = 7.88 Hz, 1H, ArH), 6.79 (d, *J* = 7.88 Hz, H, ArH), 7.09 (s, 1H, N=C-H), 9.05 (s, 1H, ArOH), 10.18 (s, 1H, OH-pyrone); ^13^C-NMR (75 MHz, (CD_3_S)_2_O) δ ppm: 19.52 (CH_3_-pyrone), 37.81 (C-3), 90.16 (C-5), 99.73, 114.89, 118.31, 120.00 ( ArC and ArCH), 123.32 (C-S thiazole), 125.19 (C-N thiazole), 127.30 (C-2), 142.78, 145.04 (ArC-OH), 161.58 (C-6), 164.02 (C=N thiazole), 167.29 (C=N), 169.25 (C-4); HRMS (ESI) *m*/*z* calc for C_16_H_13_N_2_O_5_S 345.0545, found 345.0552 [M + H]^+^.

*4-Hydroxy-6-methyl-3-(2-[(4-nitrophenyl)methylidene]amino-1,3-thiazol-4-yl)-2H-pyran-2-one* (**4d**). Yield 85%. Brown solid; m.p. 94 °C; IR neat (cm^−1^): 3050–3500, 2957, 2174, 1684, 1608, 1563, 1510, 1450, 1358, 1303, 1250, 1179, 1027, 996, 874, 827, 721. ^1^H-NMR (300 MHz, (CD_3_S)_2_O), δ ppm: 2.21 (s,3H, CH_3_), 6.05 (s, 1H, H-thiazole), 7.60 (s, 1H, H-pyrone), 8.15 (d, *J* = 8.51 Hz, 2H, ArH), 8.40 (d, *J* = 8.51 Hz, 2H, ArH), 9.21(s, 1H, OH-pyrone), 10.17(s, 1H, N=C-H);^13^C-NMR (75 MHz, (CD_3_S)_2_O), δ ppm: 19.61 (H_3_C-pyrone), 90.65 (C-3) 99.42 (C-5), 121.43 (C-S at thiazole), 123.35, 123.81, 130.61, 136.04, 142.80 (ArC and ArCH), 150.54 (C-N at thiazole), 161.60 (C-6), 164.61 (ArC-NO_2_), 167.45 (C=N at thiazole), 167.70 (C-2), 171.43 (C=N), 192.38 (C-4);HRMS (ESI) *m*/*z* calc for C_16_H_12_N_3_O_5_S 358.0498, found 358.0495 [M + H]^+^.

*3-(2-[(4-Chlorophenyl)methylidene]amino-1,3-thiazol-4-yl)-4-hydroxy-6-methyl-2H-pyran-2-one* (**4e**). Yield 87%. Orange solid; m.p. 238 °C; IR neat (cm^−1^): 3050–3500, 2955, 1992, 1693, 1618, 1489, 1450, 1354, 1322, 1207, 1172, 1038, 997, 865, 821, 779, 721. ^1^H-NMR (300 MHz, (CD_3_S)_2_O), δ ppm: 2.20 (s, 3H, CH_3_), 5.43 (s, 1H, H-pyrone), 6.03 (s, 1H, H-thiazole), 6.47 (d, *J* = 8.63 Hz, 1H, ArH), 6.68 (d, *J* = 8.63 Hz, 1H, ArH), 7.79 (d, *J* = 8.57 Hz, 1H, ArH), 7.07 (s, 1H, N=C-H), 7.12 (d, *J* = 8.57 Hz, 1H, ArH), 9.01 (s, 1H, OH-pyrone); ^13^C-NMR(75 MHz, (CD_3_S)_2_O), δ ppm:19.50 (H_3_C-pyrone), 90.55 (C-3), 99.55 (C-5), 121.51 (C-S at thiazole), 128.31, 129.11, 129.68 (ArC and ArCH), 131.10 (C-N at thiazole), 132.38 (C-6), 137.75 (ArC-Cl), 161.55 (C=N at thiazole), 164.31 (C-2), 167.35 (C=N), 169.24 (C-4); HRMS (ESI) *m*/*z* calc for C_16_H_12_ClN_2_O_3_S 347.0257, found 347.0256 [M + H]^+^.

*4-Hydroxy-3-(2-[(4-hydroxyphenyl)methylidene]amino-1,3-thiazol-4-yl)-6-methyl-2H-pyran-2-one* (**4f**). Yield 88%. Orange solid; m.p. 129 °C; IR neat (cm^−1^): 3050–3500, 2957, 1717, 1683, 1621, 1512, 1450, 1359, 1285, 1213, 1175, 1037, 996, 859, 835, 723. ^1^H-NMR (300 MHz, (CD_3_S)_2_O), δ ppm: 2.21 (s, 3H, CH_3_), 5.41 (s, 1H, H-pyrone), 6.06 (s, 1H, H-thiazole), 6.69 (d, *J* = 8.7 Hz, 1H, ArH), 6.92 (d, *J* = 8.4 Hz, 1H, ArH), 7.07 (s, 1H, N=C-H), 7.04 (d, *J* = 8.4 Hz, 1H, ArH), 7.74 (d, *J* = 8.7 Hz, 1H, ArH), 9.17 (s, 2H, ArOH), 9.79 (s, 1H, OH-pyrone); ^13^C-NMR (75 MHz, (CD_3_S)_2_O), δ ppm: 19.50 (H_3_C-pyrone), 90.55 (C-3), 99.55 (C-5), 121.51 (C-S at thiazole), 128.31, 129.11, 129.68 (ArC and ArCH), 131.10 (C-N at thiazole), 132.38 (C-6), 137.75 (ArC-Cl), 161.55 (C=N at thiazole), 164.31 (C-2), 167.35 (C=N), 169.24 (C-4); HRMS (ESI) *m*/*z* calc for C_16_H_13_N_2_O_4_S 329.0596, found 329.0595 [M + H]^+^.

*3-(2-[(2,3-Dihydroxyphenyl)methylidene]amino-1,3-thiazol-4-yl)-4-hydroxy-6-methyl-2H-pyran-2-one* (**4g**). Yield 82%. Brown solid; m.p. 134 °C; IR neat (cm^−1^): 3050–3500, 2958, 2836, 1719, 1683, 1621, 1510, 1450, 1358, 1303, 1215, 1179, 1023, 995, 858, 832, 721. ^1^H-NMR (300 MHz, (CD_3_S)_2_O), δ ppm: 2.21 (s, 3H, CH_3_), 5.41 (s, 1H, H-pyrone), 6.06 (s, 1H, H-thiazole), 6.69 (d, *J* = 8.75 Hz, 1H, ArH), 6.92 (d, *J* = 8.39 Hz, 1H, ArH), 7.07 (s, 1H, N=C-H), 7.04 (d, *J* = 8.39 Hz, 1H, ArH), 7.74 (d, *J* = 8.75 Hz, 1H, ArH), 9.17 (s, 2H, ArOH), 9.79 (s, 1H, OH-pyrone); ^13^C-NMR (75 MHz, (CD_3_S)_2_O), δ ppm: 19.33 (H_3_C-pyrone), 90.29 (C-3), 99.49 (C-5), 115.10 (C-S at thiazole), 116.08, 123.56, 127.75, 128.55, 128.70 (ArC and ArCH), 132.04 (C-N at thiazole), 156.74 (C-6), 161.35 (ArC-OH), 163.27 (C=N at thiazole), 164.35 (C-2), 167.02 (C=N), 169.12 (C-4); HRMS (ESI) *m*/*z* calc for C_16_H_13_N_2_O_5_S 345.0545, found 345.0541 [M + H]^+^.

*4-Hydroxy-3-(2-[(2-methoxyphenyl)methylidene]amino-1,3-thiazol-4-yl)-6-methyl-2H-pyran-2-one* (**4h**). Yield 79%. Green solid; m.p. 242 °C; IR neat (cm^−1^): 3050–3500, 2957, 2838, 1717, 1682, 1610, 1510, 1451, 1358, 1303, 1208, 1163, 1028, 996, 856, 827, 721. ^1^H-NMR (300 MHz, (CD_3_S)_2_O), δ ppm: 2.17 (s, 3H, CH_3_), 3.72 (s, 3H, OCH_3_), 5.42 (s, 1H, H-pyrone), 6.05 (s, 1H, H-thiazole), 6.79 (t, *J*_1_ = 11.31 Hz, *J*_2_ = 9.77 Hz 1H, ArH), 6.92 (d, *J* = 11.31 Hz, 1H, ArH), 7.09 (s, 1H, N=C-H), 7.22 (d, *J* = 10.51 Hz, 1H, ArH), 7.67 (t, *J*_1_ = 11.31 Hz, *J*_2_ = 9.77 Hz 1H, ArH), 7.87 (s, 1H, N=C-H), 9.86 (s, 1H, OH-pyrone); ^13^C-NMR (75 MHz, (CD_3_S)_2_O), δ ppm: 19.46 (H_3_C-pyrone), 40.33 (OCH_3_), 55.16 (ArC-OCH_3_), 99.77 (C-3), 113.67 (C-5), 114.56 (C-S at thiazole), 115.66, 123.75, 129.01 (ArC and ArCH), 129.45 (C-N at thiazole), 158.55 (C=N at thiazole), 161.63 (C-2), 163.91 (C-6), 167.17 (C=N), 169.25 (C-4); HRMS (ESI) *m*/*z* calc for C_17_H_15_N_2_O_4_S 343.0753, found 343.0748 [M + H]^+^.

*4-Hydroxy-3-(2-[(4-methoxyphenyl)methylidene]amino-1,3-thiazol-4-yl)-6-methyl-2H-pyran-2-one* (**4i**). Yield 87%. Yellow solid; m.p. 204 °C; IR neat (cm^−1^): 3050–3500, 2956, 1718, 1683, 1620, 1489, 1450, 1358, 1264, 1210, 1169, 1034, 995, 870, 822, 721. ^1^H-NMR (300 MHz, (CD_3_S)_2_O), δ ppm: 2.20 (s, 3H, CH_3_), 3.71 (s, 3H, OCH_3_), 5.46 (s, 1H, H-pyrone), 6.02 (s, 1H, H-thiazole), 6.70 (d, *J* = 11.31 Hz, 2H, ArH), 7.09 (s, 1H, N=C-H), 7.17 (d, *J* = 11.31 Hz, 2H, ArH), 9.90 (s, 1H, OH-pyrone); ^13^C-NMR (75 MHz, (CD_3_S)_2_O), δ ppm: 19.59 (H_3_C-pyrone), 55.17 (OCH_3_), 62.80 (ArC-OCH_3_), 90.26 (C-3), 99.52 (C-5), 112.95 (C-S at thiazole), 119.86, 122.87, 128.08, 129.75 (ArC and ArCH), 140.03 (C-N at thiazole), 159.28 (C=N at thiazole), 161.63 (C-2), 164.61 (C-6), 167.37 (C=N), 169.09 (C-4); HRMS (ESI) *m*/*z* calc for C_17_H_15_N_2_O_4_S 343.0753, found 343.0746 [M + H]^+^.

*4-Hydroxy-3-(2-[(3-methoxyphenyl)methylidene]amino-1,3-thiazol-4-yl)-6-methyl-2H-pyran-2-one* (**4j**). Yield 85%. Orange solid; m.p. 227 °C; IR neat (cm^−1^): 3050–3500, 2956, 1718, 1683, 1620, 1489, 1450, 1358, 1264, 1210, 1169, 1034, 995, 870, 822, 721. ^1^H-NMR(300 MHz, (CD_3_S)_2_O)), δ ppm: 2.21 (s, 3H, CH_3_), 3.71 (s, 3H, OCH_3_), 5.48 (s, 1H, H-pyrone), 6.07 (s, 1H, H-thiazole), 6.78 (s, 1H, ArH), 6.84 (d, *J* = 11.31 Hz, 1H, ArH), 6.87 (d, *J* = 10.51 Hz, 1H, ArH), 7.23 (t, *J*_1_ = 11.31 Hz, *J*_2_ = 10.51 Hz, 1H, ArH), 7.28 (s, 1H, N=C-H), 9.21 (s, 1H, OH-pyrone);^13^C-NMR(75 MHz, (CD_3_S)_2_O), δ ppm: 19.51 (H_3_C-pyrone), 55.77 (OCH_3_), 90.26 (C-3), 99.52 (C-5), 112.53(C-S at thiazole), 113.98, 120.08, 122.87, 128.16, 129.42 (ArC and ArCH), 140.03 (C-N at thiazole), 158.81 (C=N and ArC-OCH_3_), 161.37 (C=N at thiazole), 164.45 (C-2), 167.29 (C-6), 169.18 (C-4);HRMS *m*/*z* calc for C_17_H_15_N_2_O_4_S 343.0753, found 343.0753 [M + H]^+^.

## 4. Pharmacological Assay

### 4.1. Antioxidant Activity Evaluation

The antioxidant capacity of compounds **4a**–**4k** was evaluated by the method of Wang et al. 52(1998) [52]. The absorbance was measured at 517 nm against a blank, i.e., without DPPH. All tests were run in triplicate and an average was used. Decrease of DPPH solution absorbance indicates an increase of DPPH radical scavenging activity. The amount of sample necessary to decrease the absorbance of DPPH by 50% (IC50) was calculated graphically and the percentage inhibition was calculated according to the equation:

$$\%\;\mathrm{inhibition}=\frac{\left(\mathrm{Ao}-\mathrm{At}\right)}{\mathrm{Ao}}\;\times \;100$$

where A_o_ is the absorbance of the control at t = 0 min; and A_t_ is the absorbance of the antioxidant at t = 30 min. The food preservative butylhydroxytoluene (BHT) was used as positive control.

### 4.2. Antibacterial Assay

Clinical isolation of two Gram-negative (*Escherichia coli* ATCC 25922 and *Pseudomonas aeruginosa* ATCC 27853) and one Gram-positive (*Staphylococcus aureus* ATCC 43300) bacteria strains was obtained from the Laboratory of Cellular and Molecular Biology, Faculty of Biological Sciences, Houari Boumediene University of Sciences and Technology (USTHB), Algeria. The minimum inhibitory concentration (MIC) values were evaluated using the serial dilution method according to standard methods (NCCLS, 532003) [53]. Stock solutions of the compounds **4a**–**4k** and antibiotic standards (amoxicillin and ciprofloxacin) were prepared in DMSO. Dilution series, using MHB, were prepared from 10 to 200 μg/mL. After incubation at 37 °C for 24 h, the microorganism growth inhibition was evaluated by measuring absorbance at 630 nm, using a spectrophotometer. Experiments were performed in triplicate at three different times.

## 5. Conclusions

In conclusion, an efficient and simple one-pot multi-component condensation procedure was introduced and developed for the synthesis of new thiazole derivatives, under conventional heating or under ultrasound irradiation, using SiW.SiO_2_ as a reusable catalyst. The present method is bestowed with several advantages, such as an inexpensive and efficient catalyst, high reaction rate, high yield, simple workup procedure and high regioselectivity. Utilizing ultrasonic irradiation techniques provided dramatic improvements in terms of higher yields and shorter reaction times compared with the conventional heating method. This procedure would be a valuable addition to the current methodologies.

The preliminary studies of these compounds proved that the thiazole ring enhances the antibacterial as well asantioxidantactivities of the synthesis thiazoles, which might serve as new templates in the synthesis and development of potent therapeutics.

## Figures and Tables

**Table 1:** Screening of the amount of catalyst, solvents, and temperature in the one-pot synthesis of Hantzsch thiazole derivative **4a**.

Entry	Solvent	Catalysis (%)	Time (h)	Temp (°C)	Yield (%)
1	Water	15	24	RT	10
2	Water	15	6	100	45
3	MeOH	15	24	RT	18
4	MeOH	15	4	60	55
5	EtOH	15	24	RT	60
6	EtOH	15	2	65	87
7	1-Butanol	15	6	110	60
8	2-propanol	15	3	80	65
9	EtOH/Water	15	24	RT	70
10	EtOH/Water	15	2	65	87
11	EtOH/Water	-	7	65	50
12	EtOH/Water	5	2	65	74
13	EtOH/Water	10	2	65	79
14	EtOH/Water	18	2	65	87
15	EtOH/Water	20	2	65	87

**Table 2:** Synthesis of Hantzsch thiazole derivatives under conventional heating and under ultrasonic irradiation.

Product	Substitutions			Method A ^a^		Method B ^b^	
	R_1_	R_2_	R_3_	Time (h)	Yield (%)	Time (h)	Yield (%)
**4a**	H	H	H	2	87	1.5	90
**4b**	H	OH	H	2	85	1.5	88
**4c**	OH	H	OH	3.5	80	2	82
**4d**	NO_2_	H	H	2	82	1.5	85
**4e**	Cl	H	H	2	84	1.5	87
**4f**	OH	H	H	2	85	1.5	88
**4g**	H	OH	OH	3.5	79	2	82
**4h**	H	H	OCH_3_	3.5	75	2	79
**4i**	OCH_3_	H	H	2	84	1.5	87
**4j**	H	OCH_3_	H	2	82	1.5	85

^a^ Conventional heating, 65 °C, ETOH/Water (50/50). ^b^ Ultrasonic irradiation, RT, ETOH/Water (50/50).

## Supplementary Materials

Supplementary materials are available online.
