# Supplementary material for: Synthesis and Biological Evaluation of New Substituted Hantzsch Thiazole Derivatives from Environmentally Benign One-Pot Synthesis Using Silica Supported Tungstosilisic Acid as Reusable Catalyst
Source: Molecules. 2017 May 7;22(5):757. doi: 10.3390/molecules22050757 (PMC6153747; doi:10.3390/molecules22050757)
Supplement: Supplementary file 1 [file molecules-22-00757-s001.pdf]

Synthesis and biological evaluation of new substituted Hantzsch thiazole derivatives from environmentally benign one-pot synthesis using silica supported tungstosilisic acid as reusable catalyst

Houria Bouherrou[a], Aicha Saidoun[a], Ahmed Abderrahmani[b], Lamia Abdellaziz[b],Yahia Rachedi[a*], Françoise Dumas[c] and Albert Demenceau[d].

[a]Laboratory of Applied Organic Chemistry, Faculty of Chemistry, University of Science and Technology Houari Boumediene, BP 32, El-Alia, 16111 Bab-Ezzouar, Algiers, Algeria.

[b]Laboratory of Cellular and Molecular Biology, Faculty of Biological Sciences, USTHB, BP 32, El Alia, 16111, Bab Ezzouar, Algiers, Algeria.

[c] Chemistry of Natural Substances and Pharmacognosy, Laboratory BioCIS UMR CNRS, IPSIT and LabEx LERMIT, Faculté de Pharmacie, Université Paris Sud; 5, rue Jean Baptiste Clément, F-92296 Châtenay-MalabryCedex, France.

[d] Laboratory of Macromolecular Chemistry and Organic Catalysis, Institut de Chimie (B6a), Université de Liège, Sart-Tilman par 4000 Liège, Belgium.

** Corresponding author. Fax: +213 (0)21247311; Tel: +213 (0)21247311, email: email: hbouherrou@gmail.com*

Sommaire:

- ^{1}H NMR SPECTRA OF COMPOUND4a..3
- ^{13} C NMR SPECTRA OF COMPOUND 4a...3
- MASS SPECTRA OF COMPOUND 4a..4
- ^{1}H NMR SPECTRA OF COMPOUND 4b..4
- ^{13} C NMR SPECTRA OF COMPOUND 4b...5
- MASS SPECTRA OF COMPOUND 4b..5
- ^{1}H NMR SPECTRA OF COMPOUND 4c..6
- ^{13} C NMR SPECTRA OF COMPOUND 4c..6
- MASS SPECTRA OF COMPOUND 4c..7
- ^{1}H NMR SPECTRA OF COMPOUND 4d..7
- ^{13} C NMR SPECTRA OF COMPOUND 4d..8
- MASS SPECTRA OF COMPOUND 4d..8
- ^{1}H NMR SPECTRA OF COMPOUND 4e...9
- ^{13} C NMR SPECTRA OF COMPOUND 4e...9
- MASS SPECTRA OF COMPOUND 4e..10
- ^{1}H NMR SPECTRA OF COMPOUND 4f..10
- ^{13} C NMR SPECTRA OF COMPOUND 4f...11
- MASS SPECTRA OF COMPOUND 4f..11
- ^{1}H NMR SPECTRA OF COMPOUND 4g..12
- ^{13} C NMR SPECTRA OF COMPOUND 4g..12
- MASS SPECTRA OF COMPOUND 4g..13
- ^{1}H NMR SPECTRA OF COMPOUND 4h..13
- ^{13} C NMR SPECTRA OF COMPOUND 4h..14
- MASS SPECTRA OF COMPOUND 4h..14
- ^{1}H NMR SPECTRA OF COMPOUND 4i..15
- ^{13} C NMR SPECTRA OF COMPOUND 4i..15
- MASS SPECTRA OF COMPOUND 4i..16
- ^{1}H NMR SPECTRA OF COMPOUND 4j...16
- ^{13} C NMR SPECTRA OF COMPOUND 4j..17
- MASS SPECTRA OF COMPOUND 4j..17
- HSQC OF COMPOUND 4f..18
- HMBC OF COMPOUND 4f..22

^{1}H NMR SPECTRA OF COMPOUND 4a:

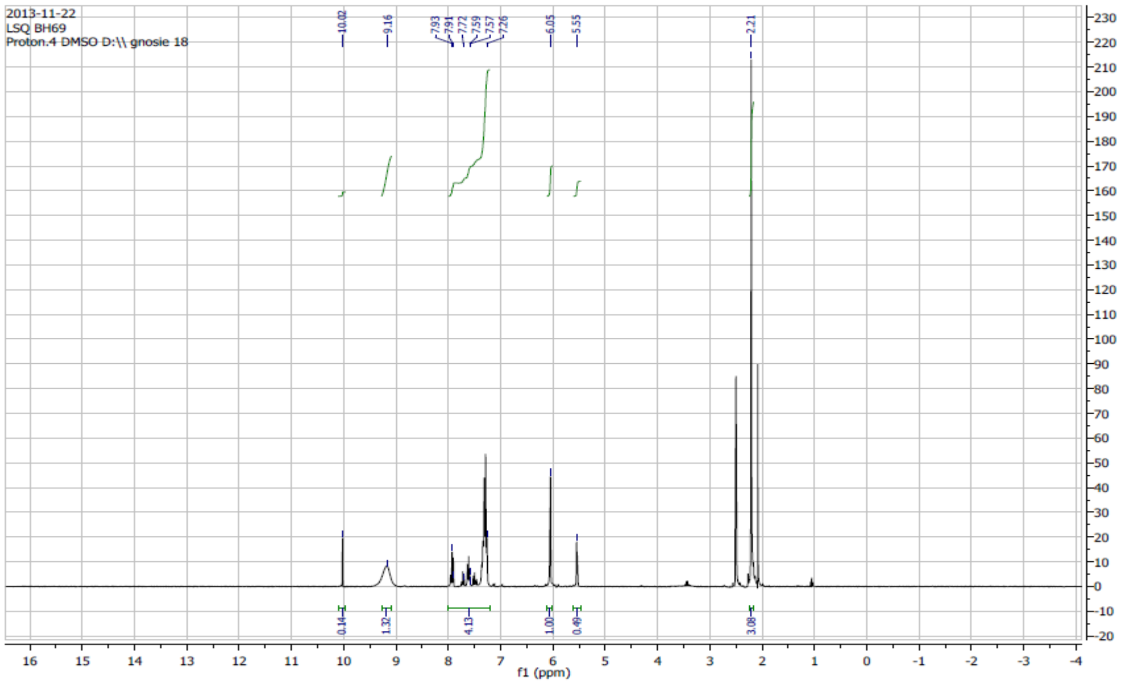

[13] C NMR SPECTRA OF COMPOUND 4a:

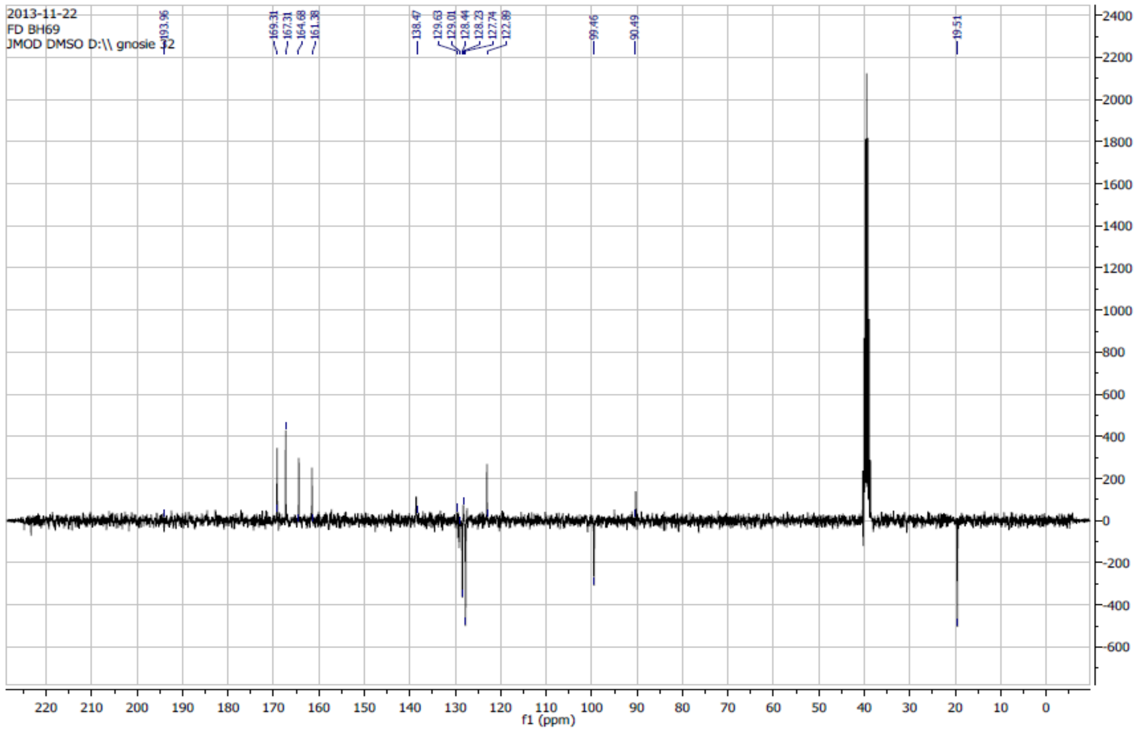

MASS SPECTRA OF COMPOUND 4a:

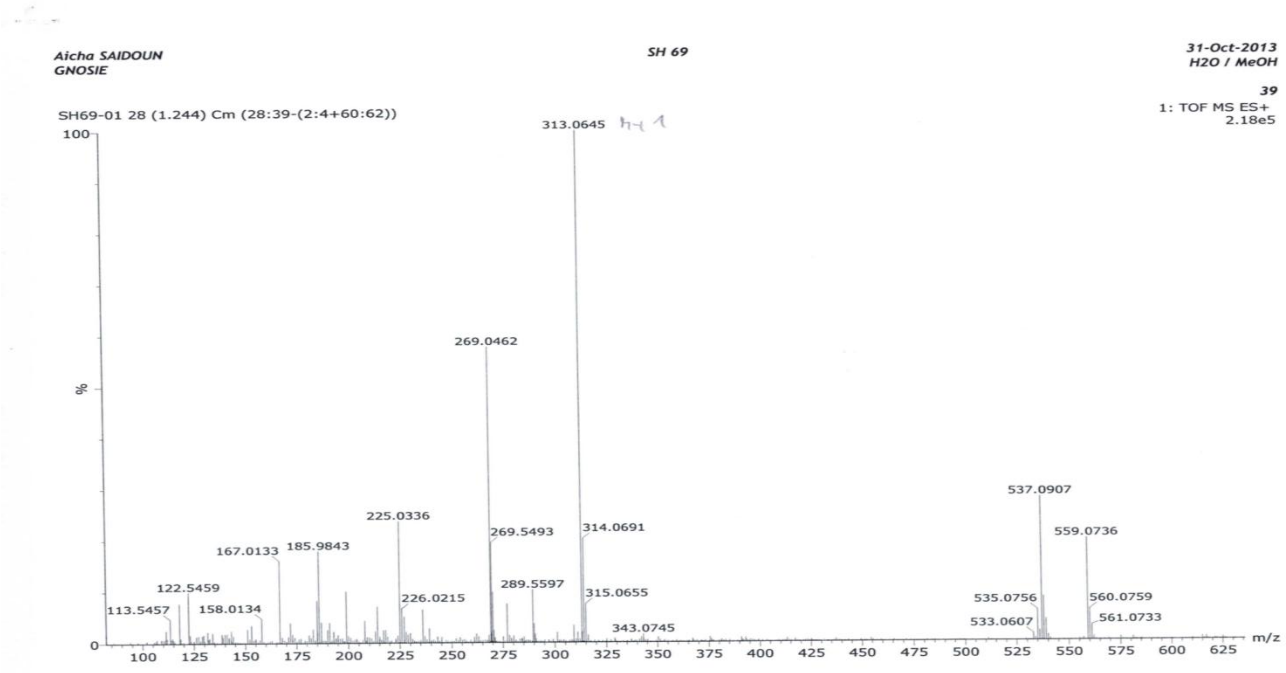

^{1}H NMR SPECTRA OF COMPOUND 4b:

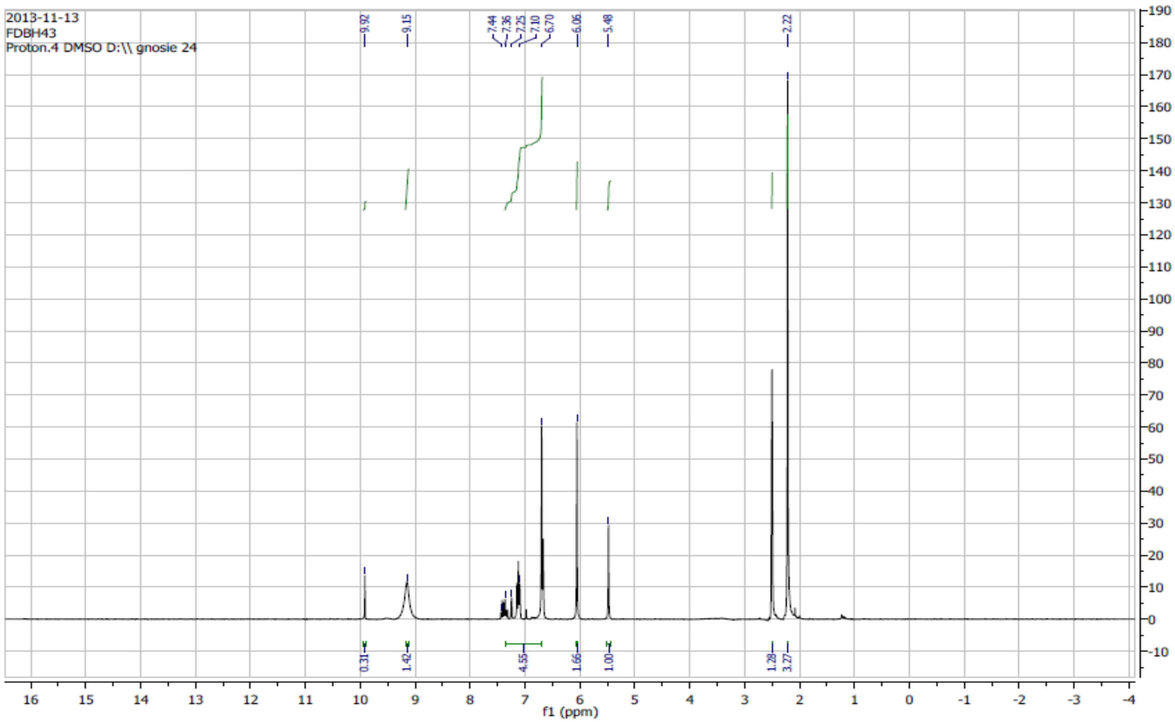

^{13}C NMR SPECTRA OF COMPOUND 4b:

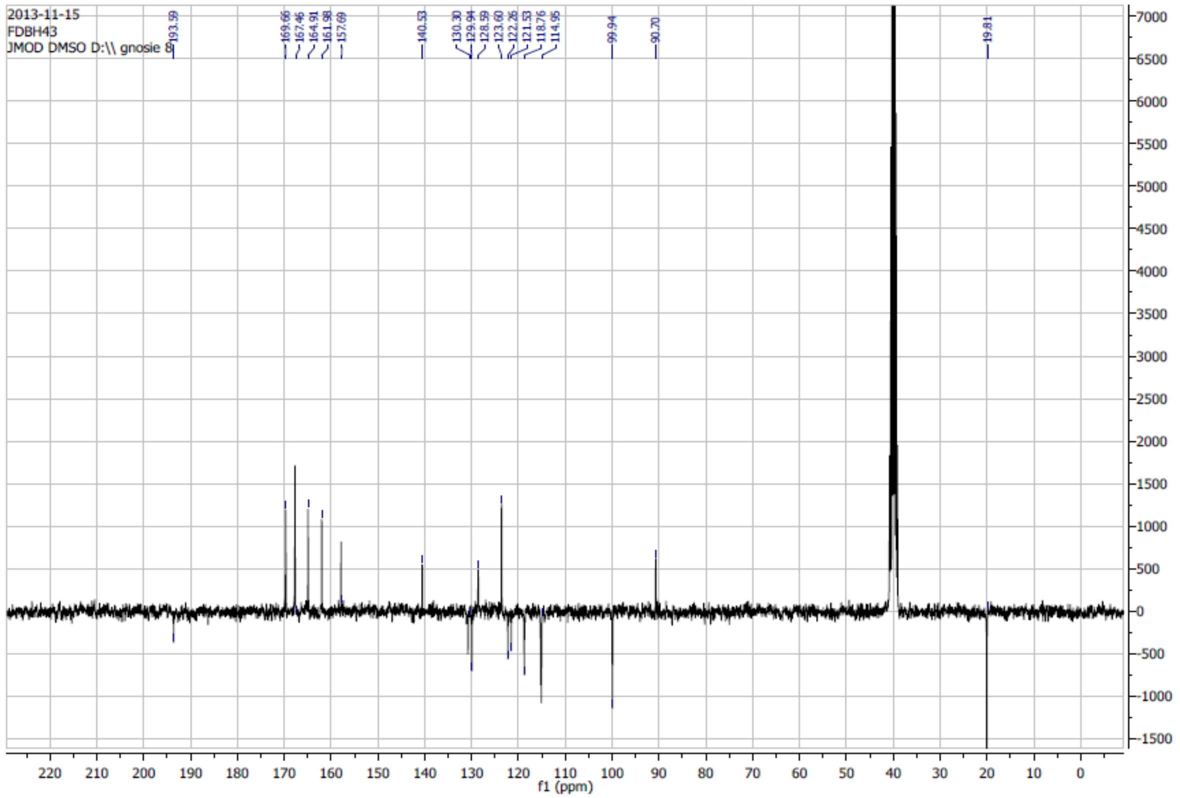

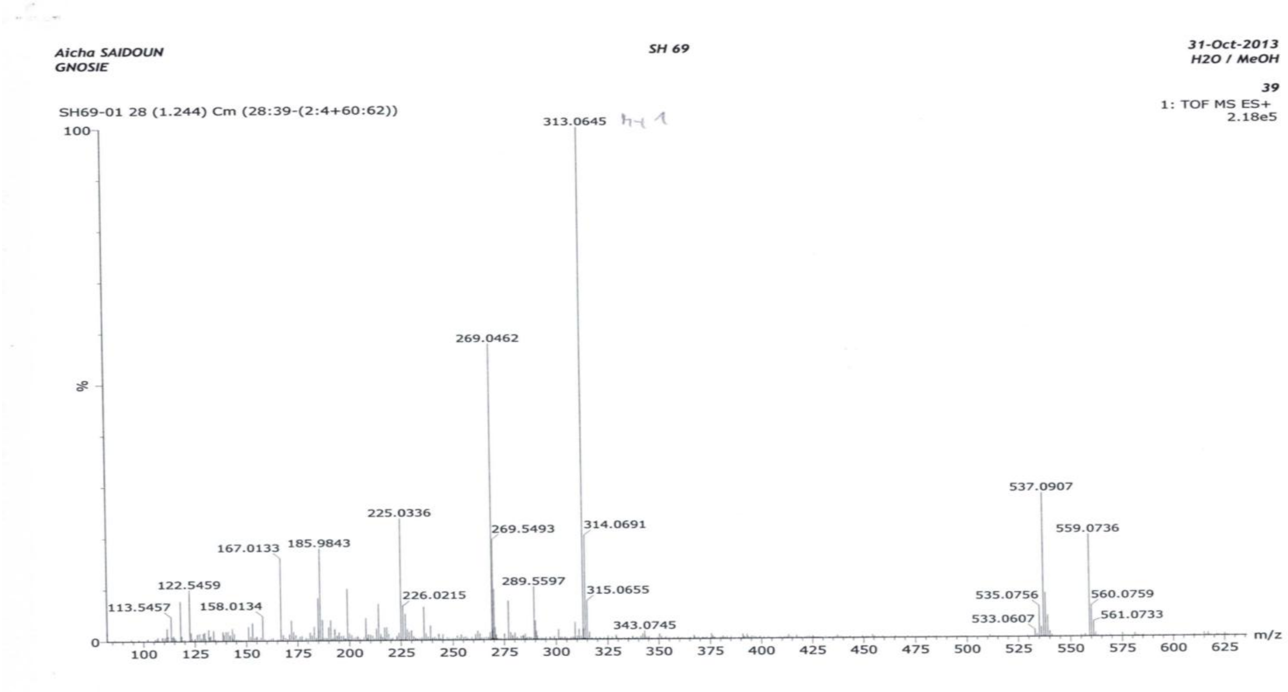

MASS SPECTRA OF COMPOUND 4b:

1 H NMR SPECTRE OF COMPOUND 4c:

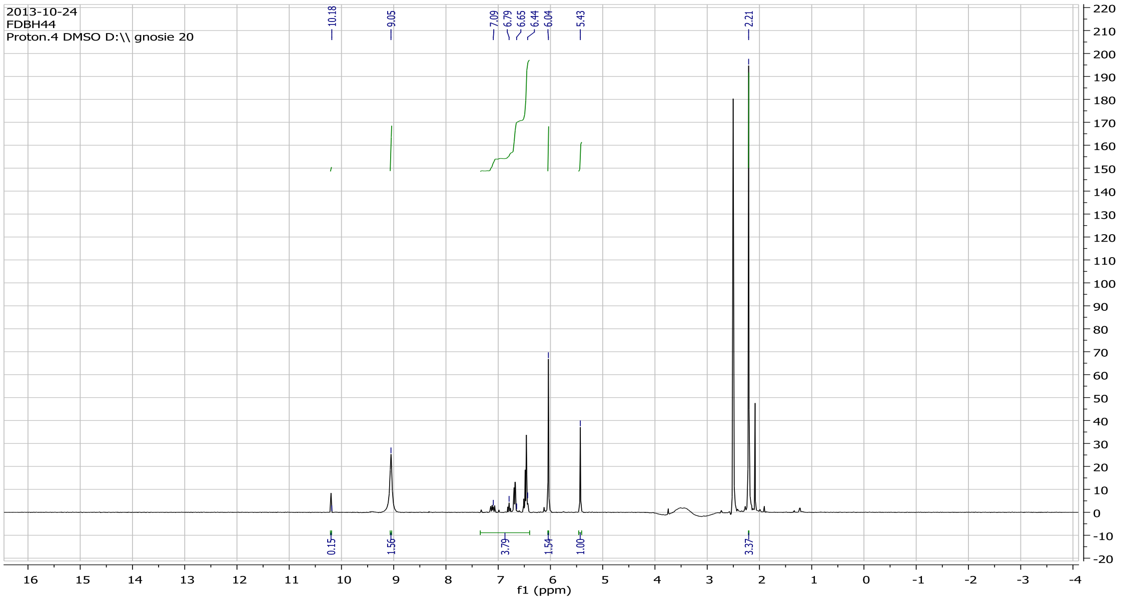

13 C NMR SPECTRE OF COMPOUND 4c:

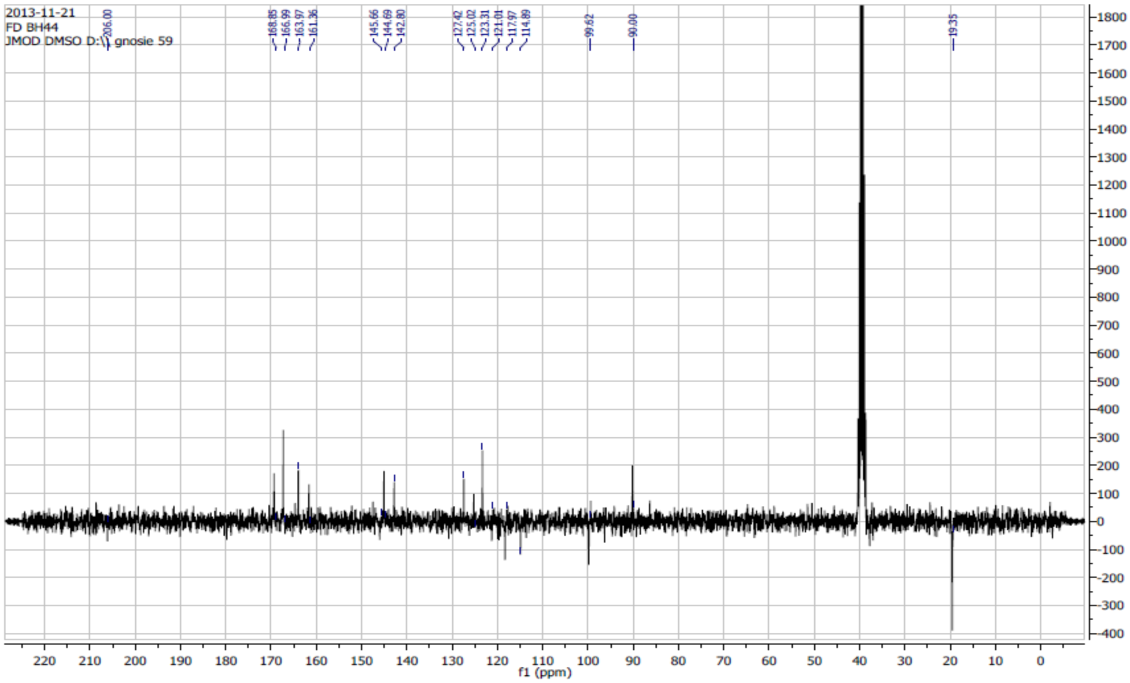

MASS SPECTRA OF COMPOUND 4c:

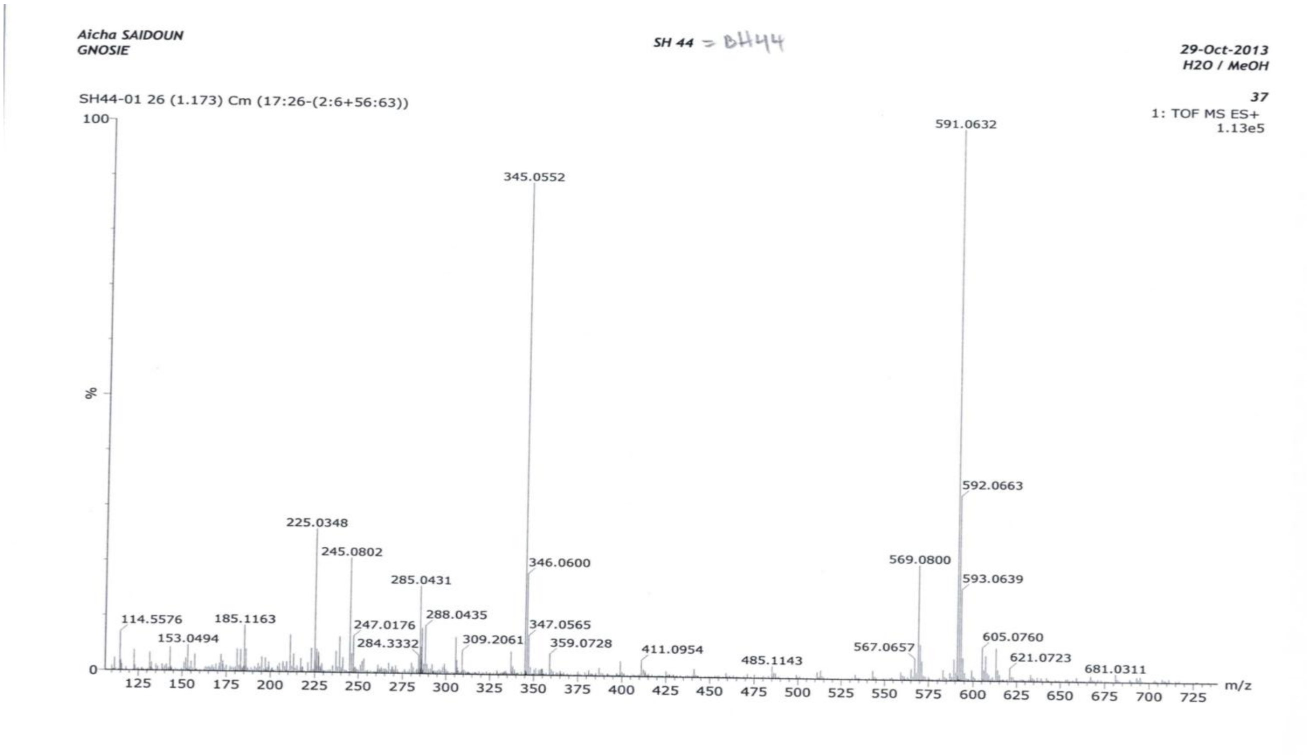

[1] H NMR SPECTRA OF COMPOUND 4d:

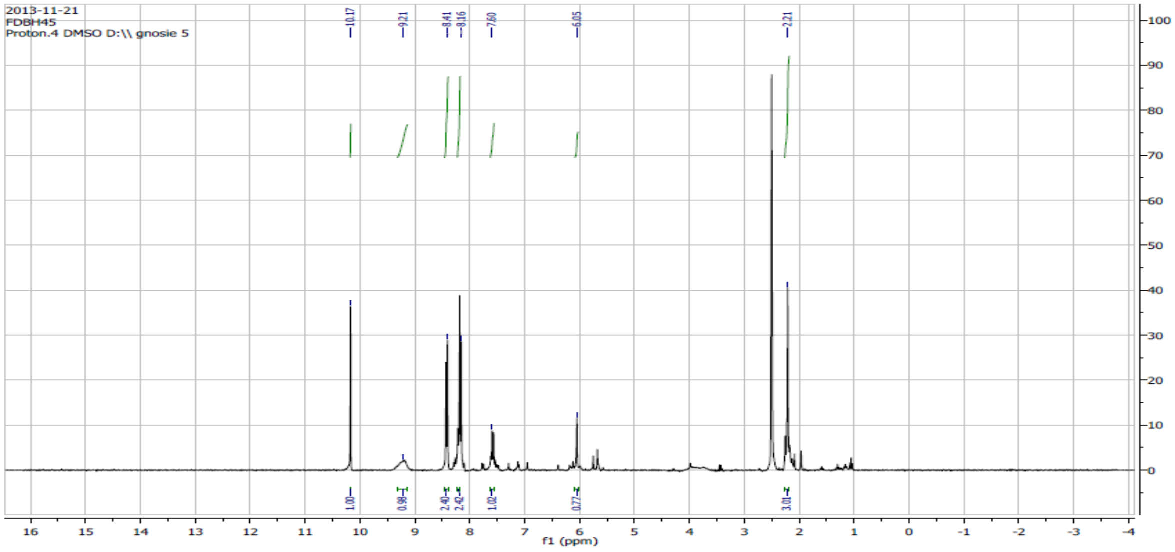

^{13}C NMR SPECTRA OF COMPOUND 4d:

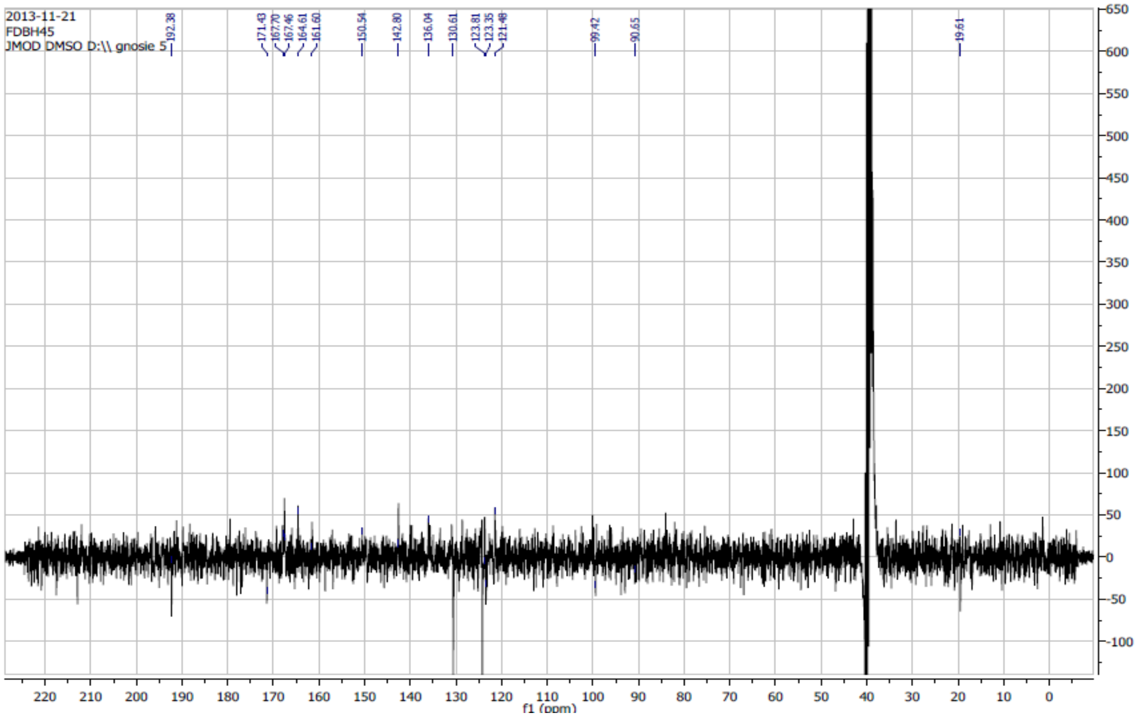

MASS SPECTRA OF COMPOUND 4d:

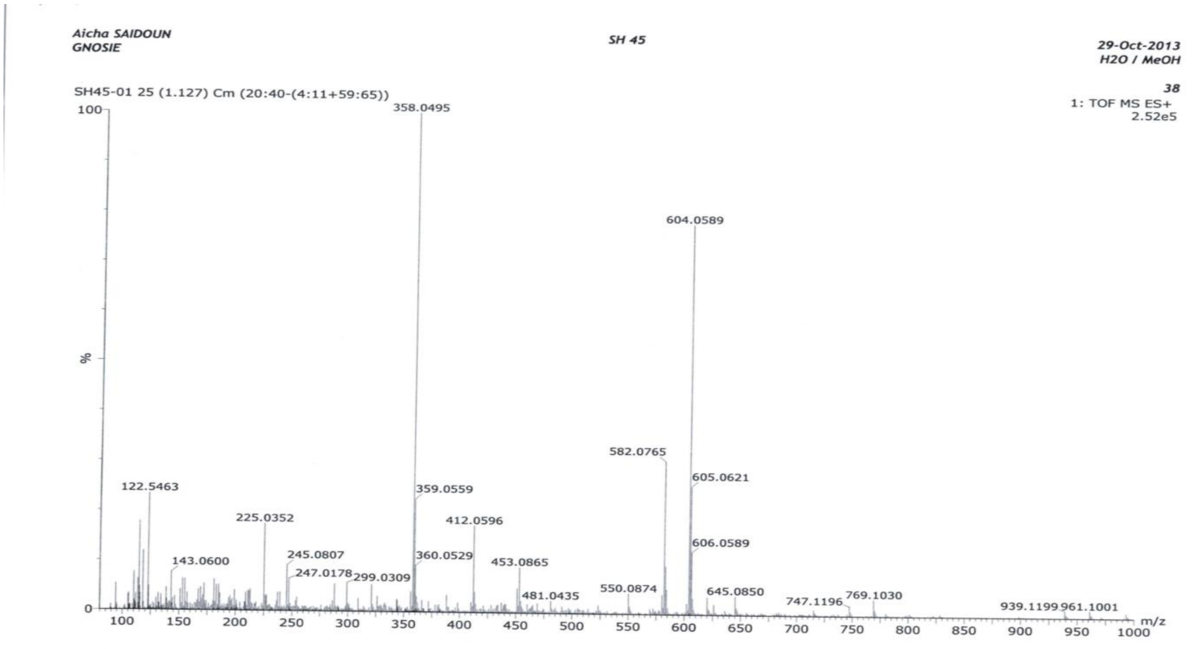

[1] H NMR SPECTRA OF COMPOUND 4e:

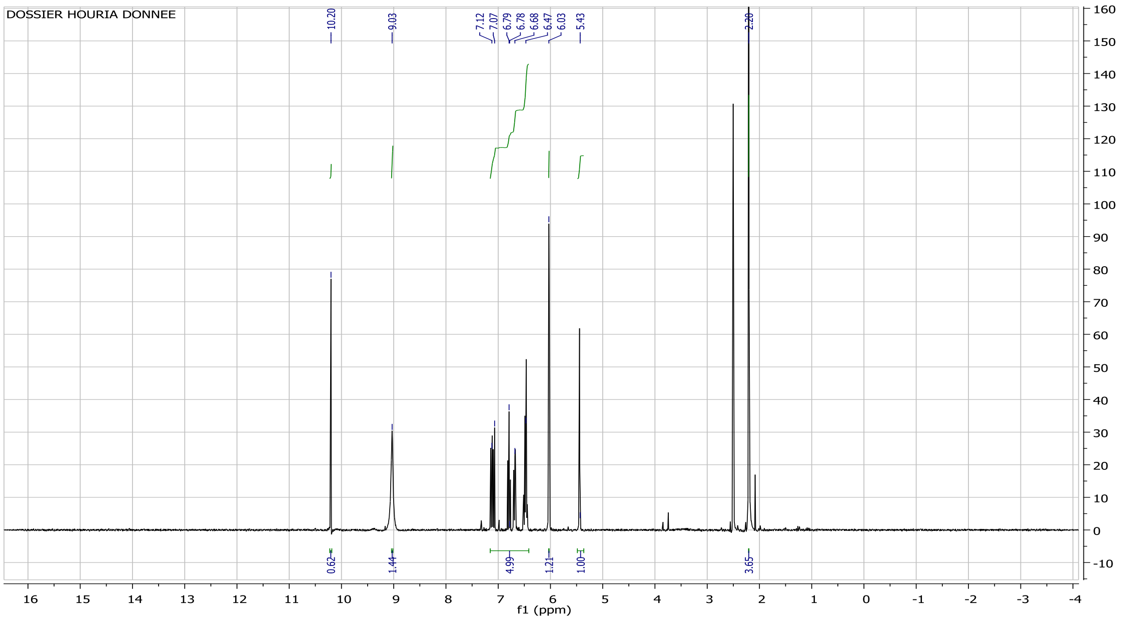

[13] C NMR SPECTRA OF COMPOUND 4e:

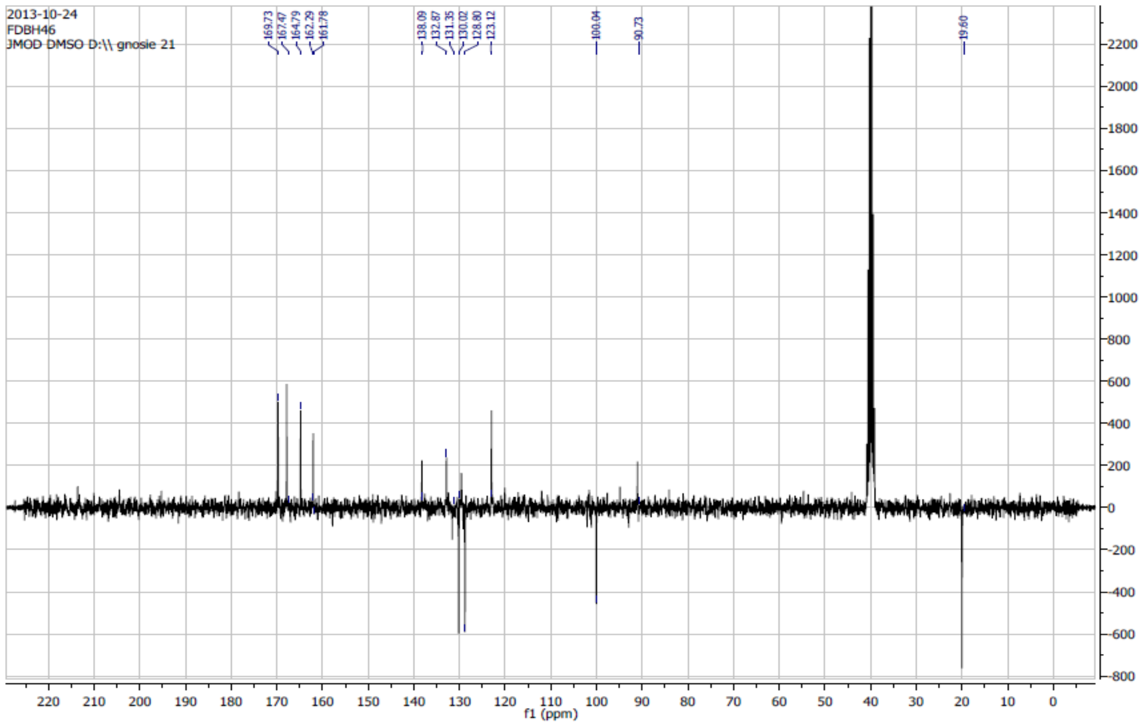

MASS SPECTRA OF COMPOUND 4e:

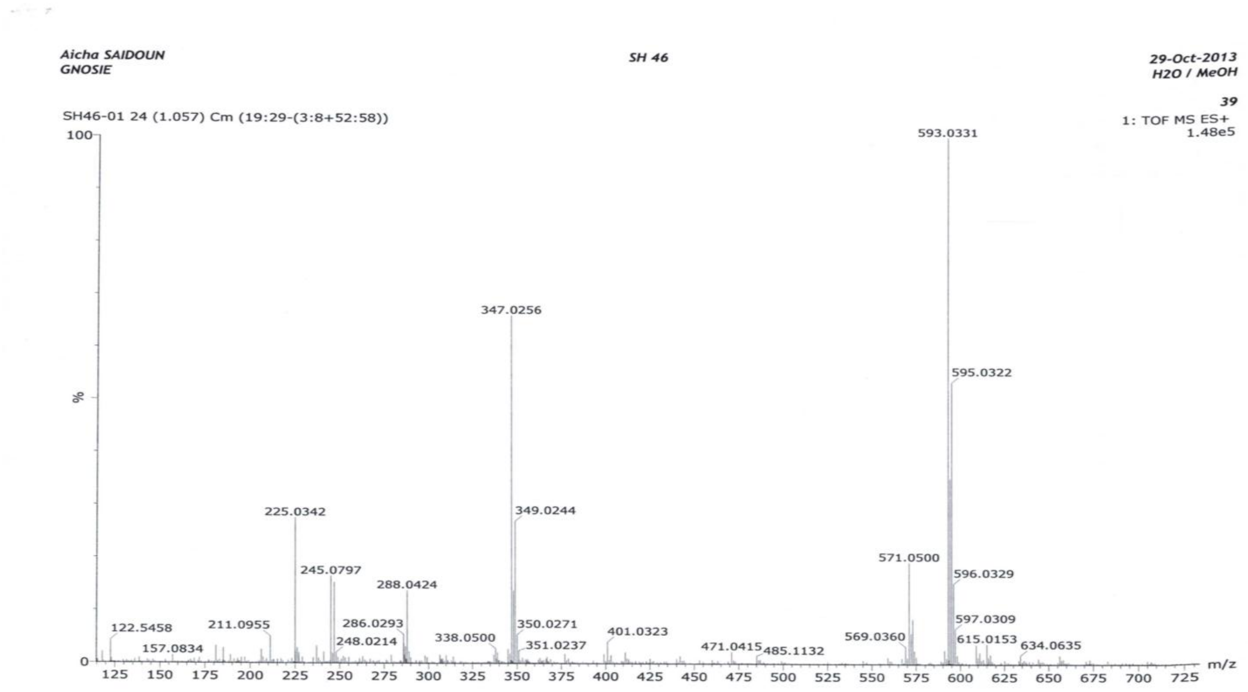

[1] H NMR SPECTRA OF COMPOUND 4f:

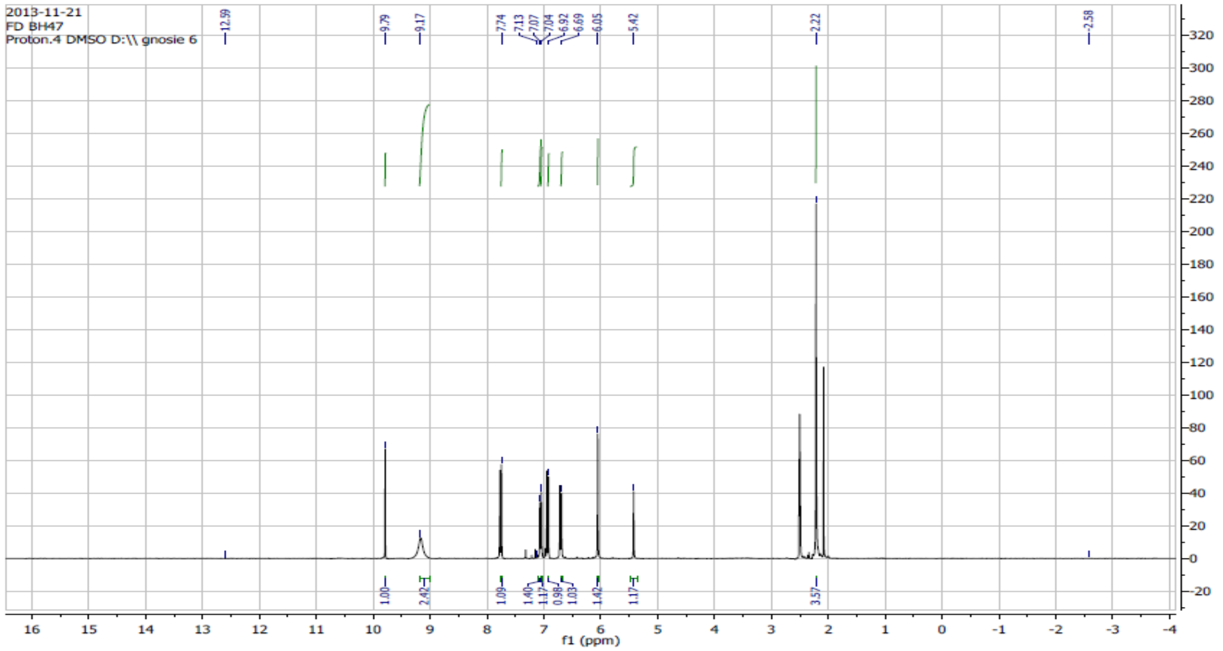

^{13}C NMR SPECTRA OF COMPOUND 4f:

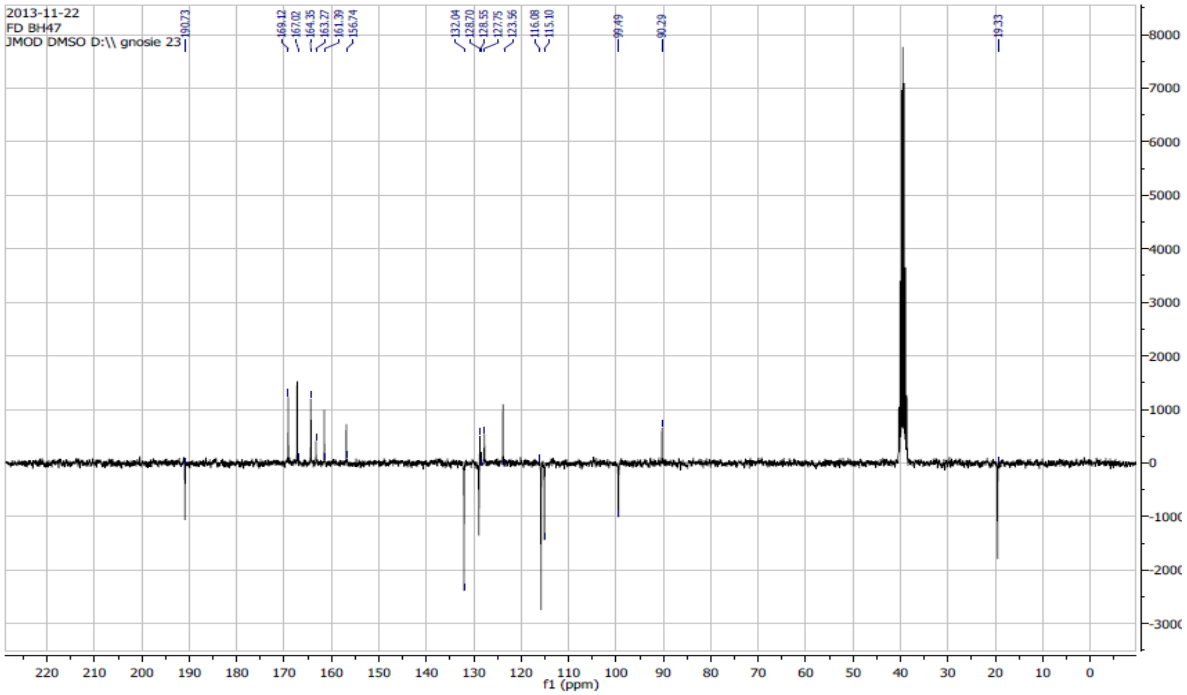

MASS SPECTRA OF COMPOUND 4f:

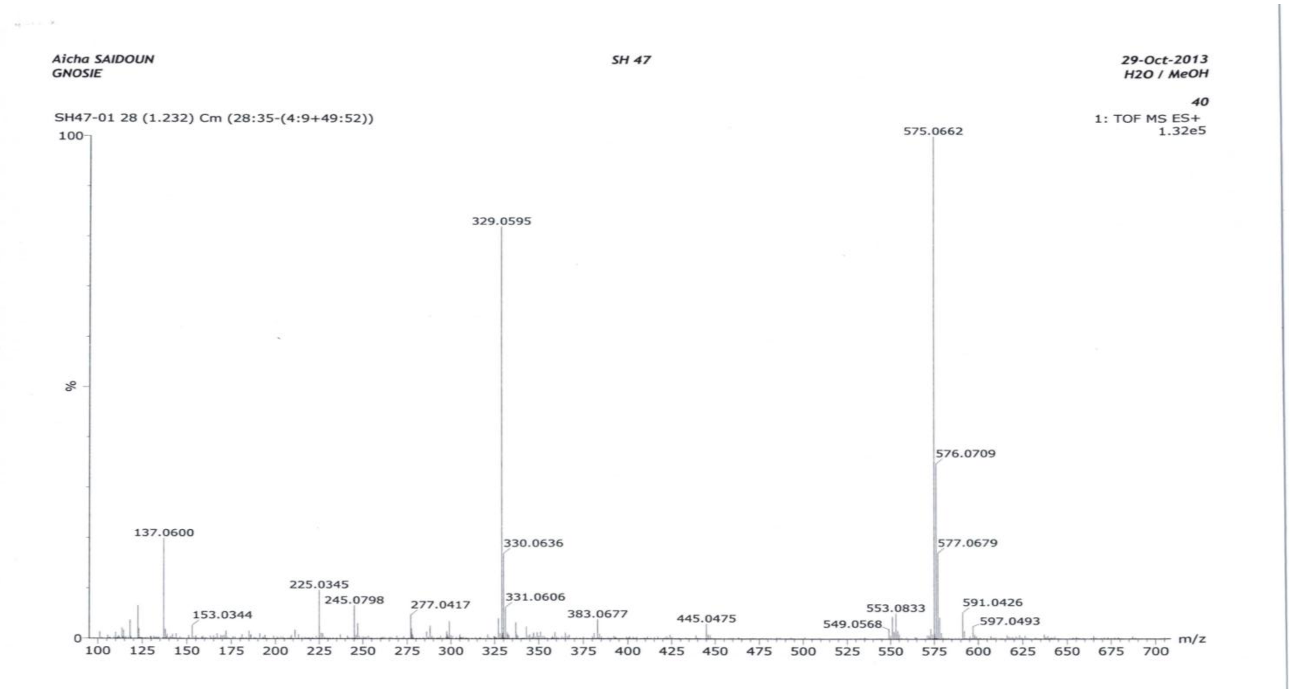

[1] H NMR SPECTRA OF COMPOUND 4g:

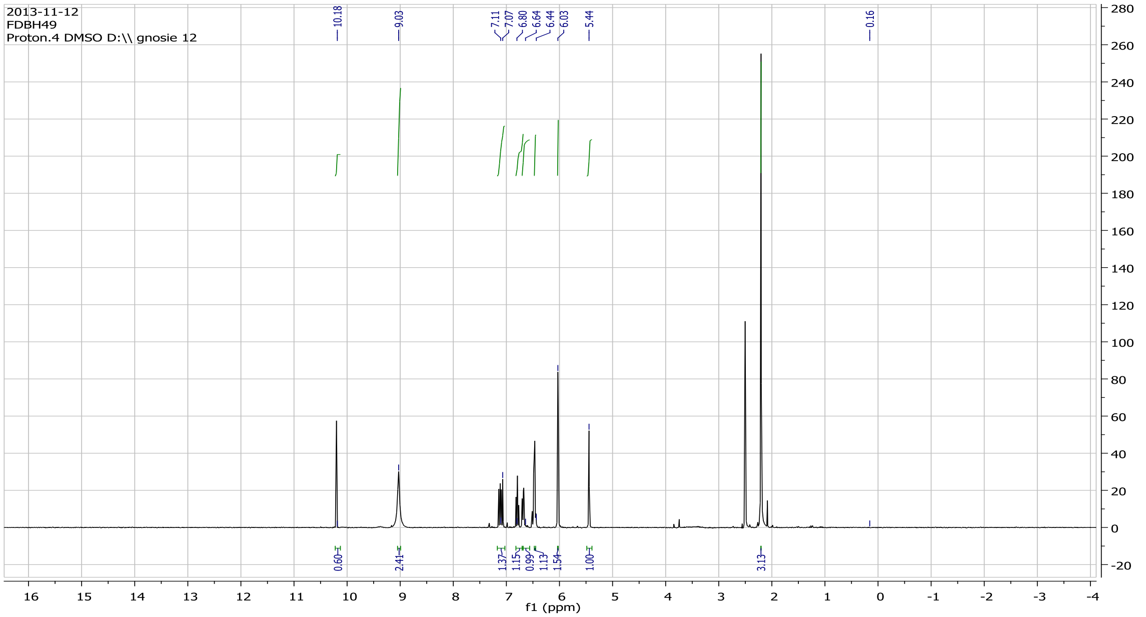

[13] C NMR SPECTRA OF CMPOUND 4g:

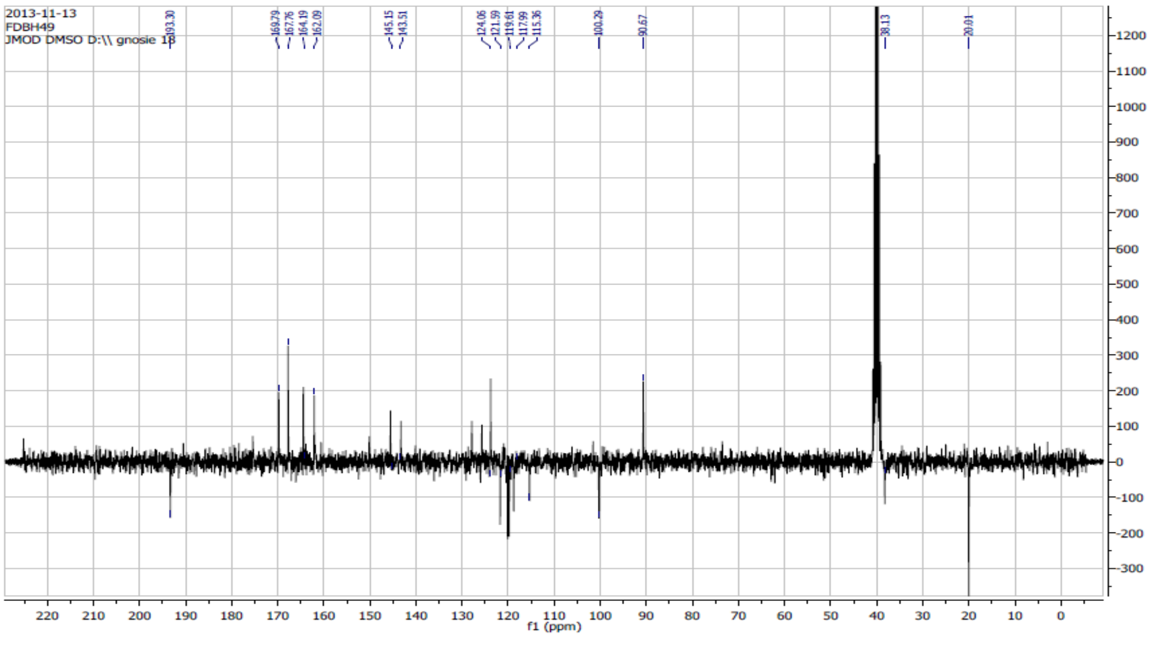

MASS SPECTRA OF COMPOUND 4g:

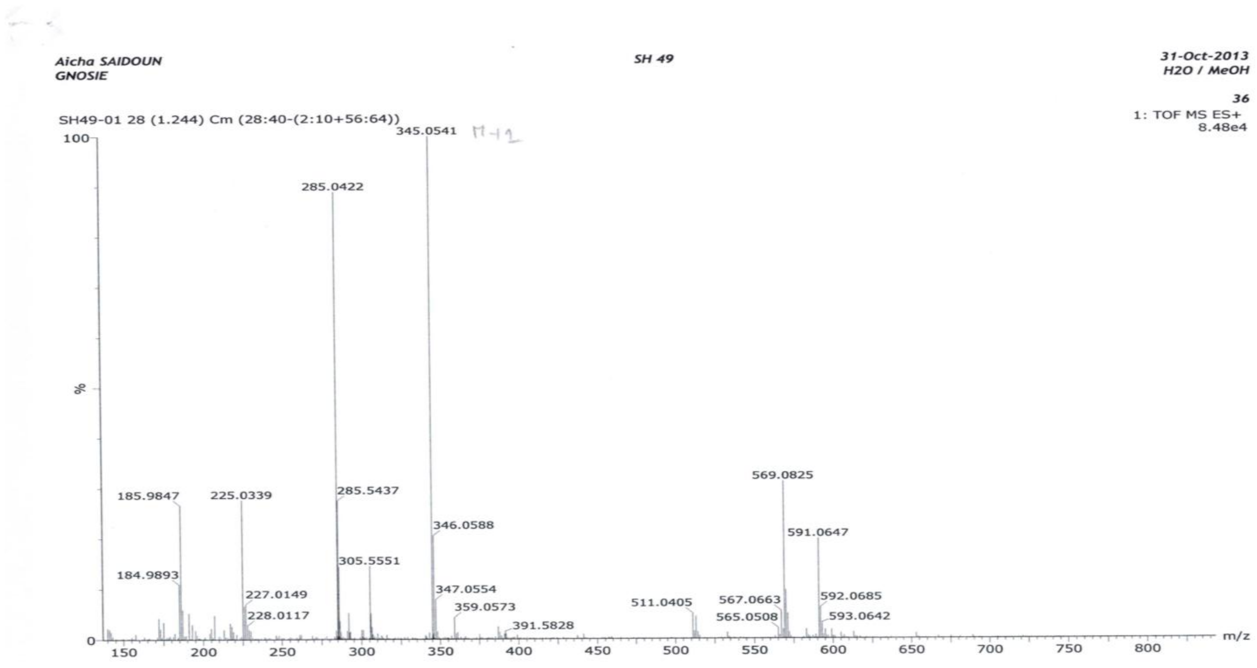

[1] H NMR SPECTRA OF COMPOUND 4h:

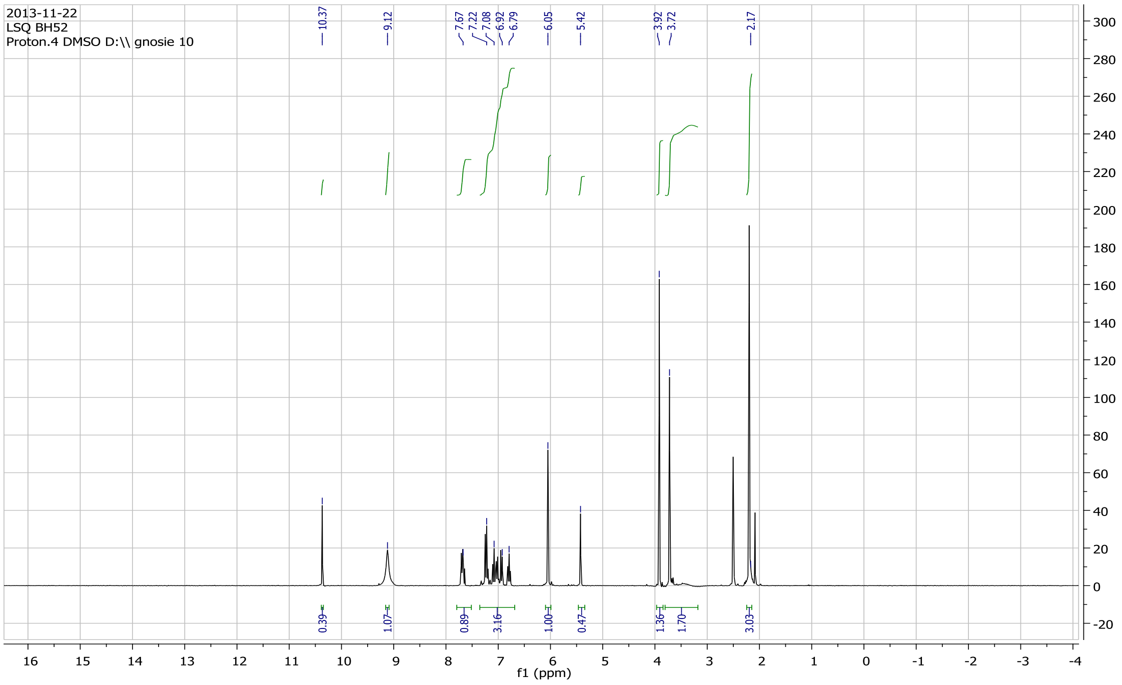

^{13}C NMR SPECTRA OF COMPOUND 4h:

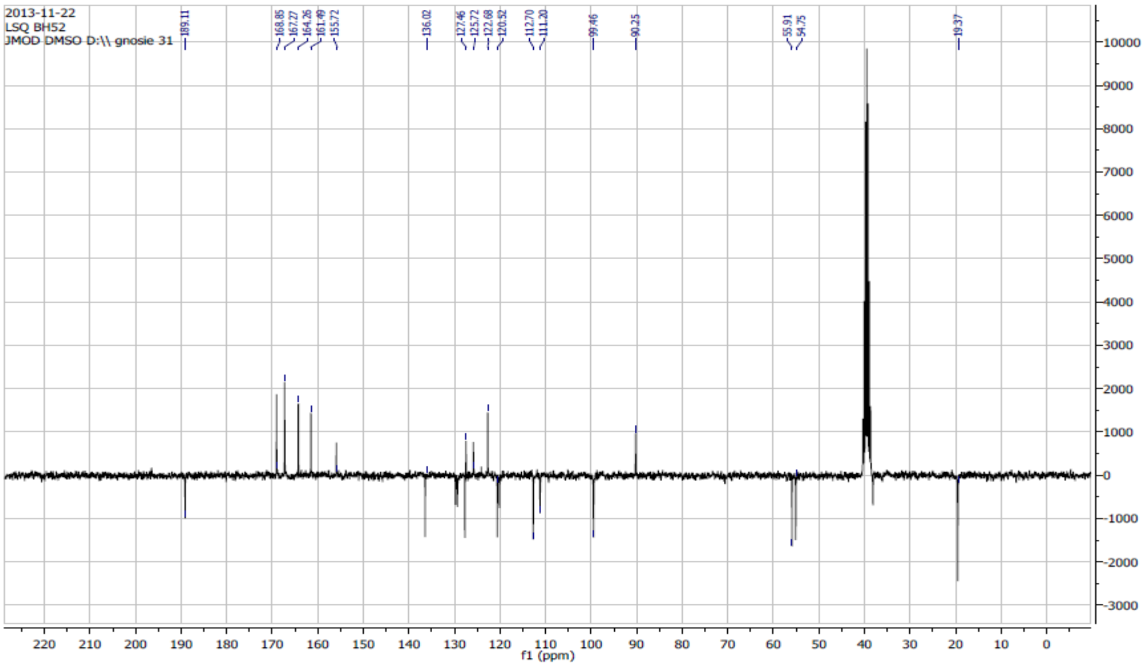

MASS SPECTRA OF COMPOUND 4h:

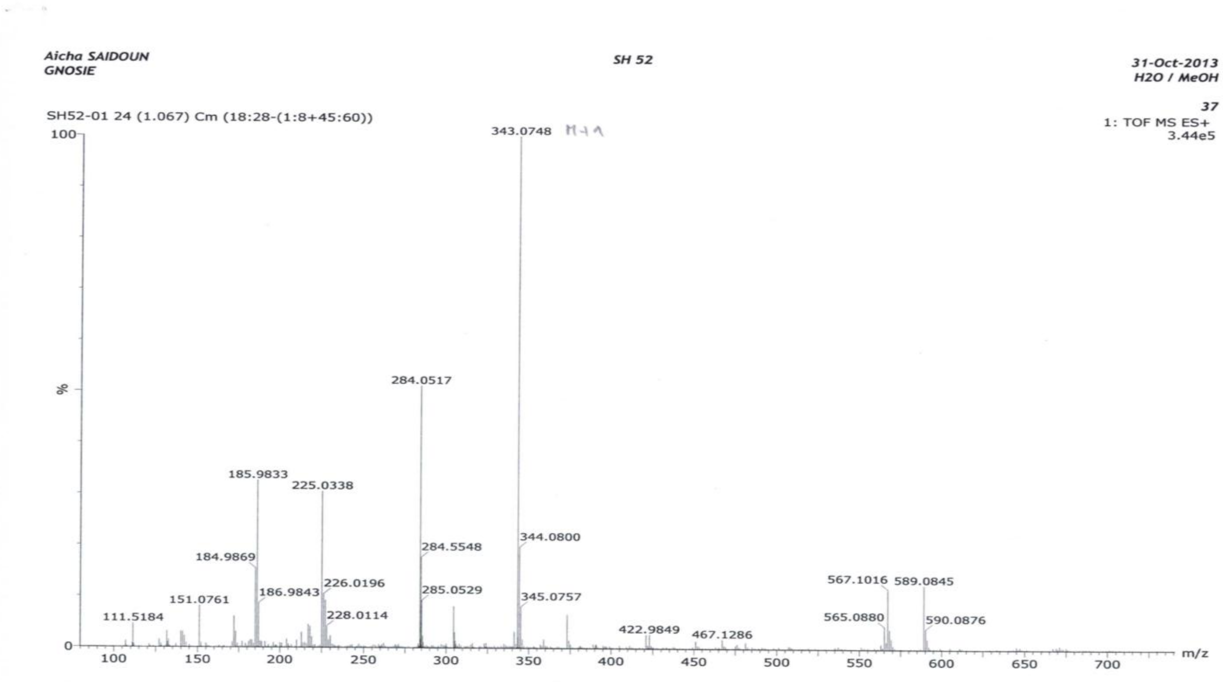

[1] H NMR SPECTRA OF COMPOUND 4i:

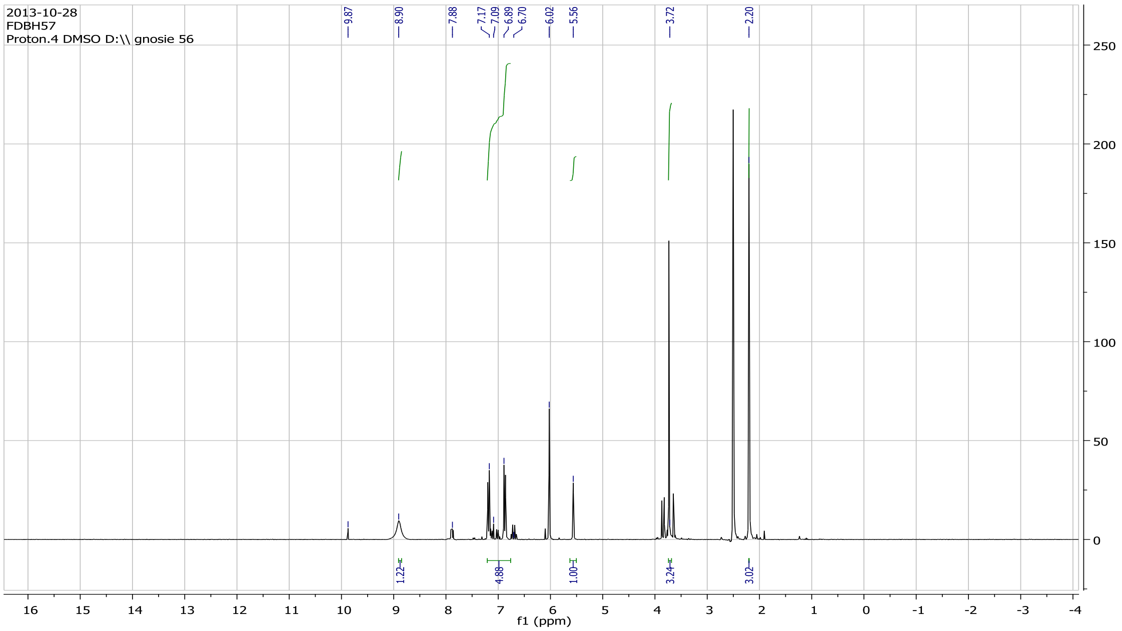

[13] C NMR SPECTRA OF COMPOUND 4i:

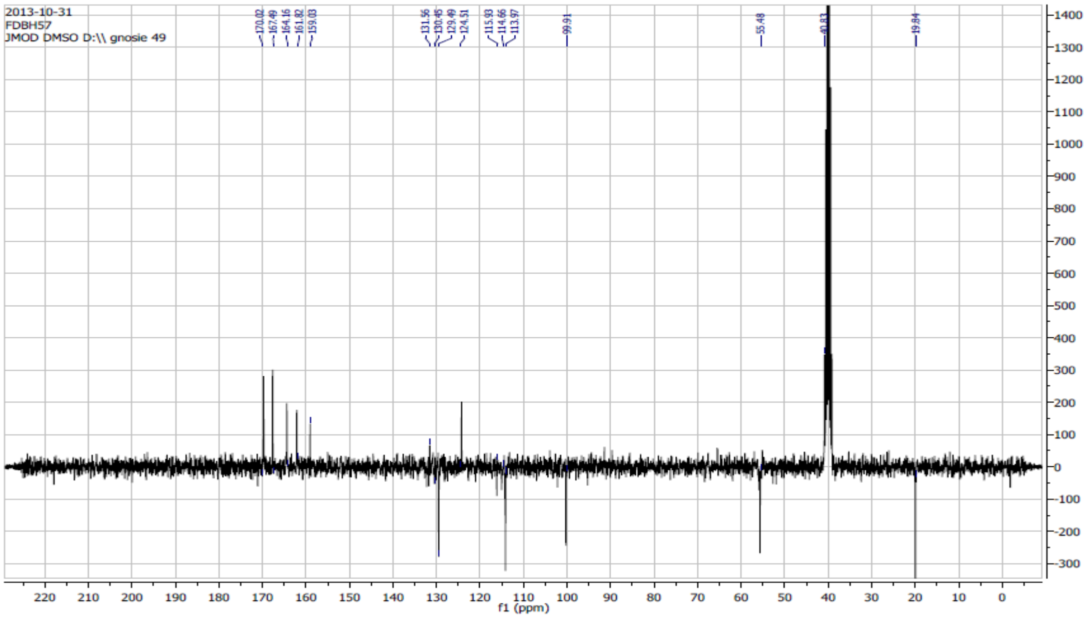

MASS SPECTRA OF COMPOUND 4i:

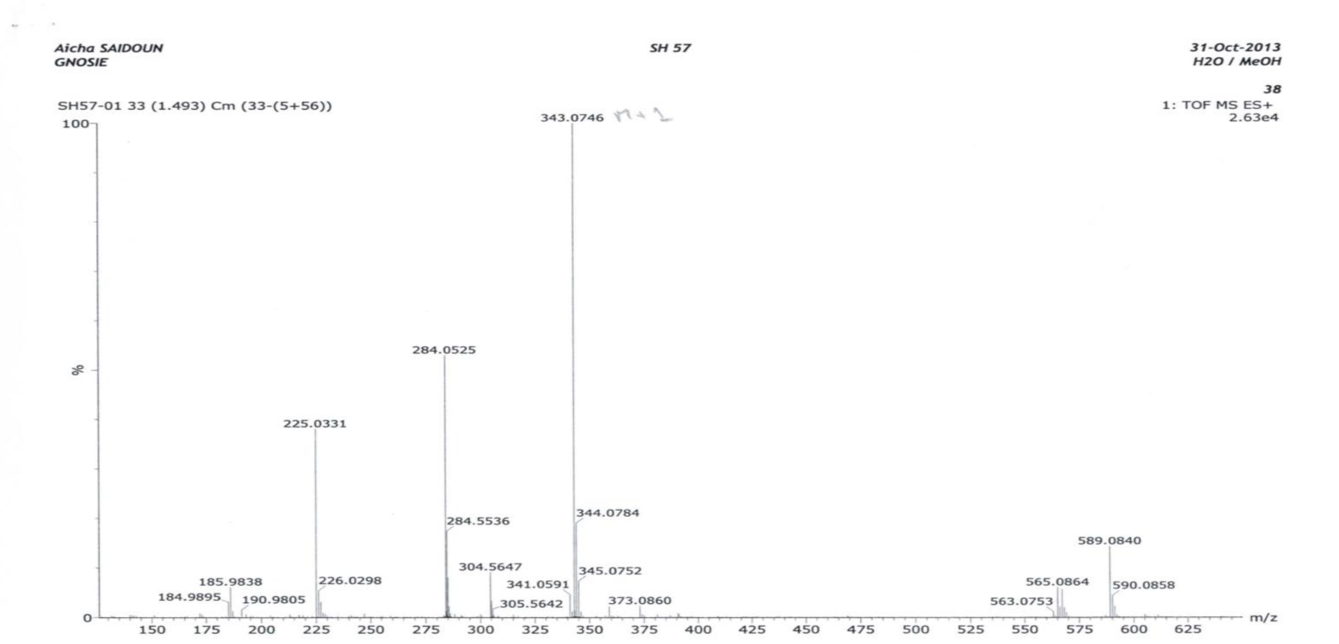

[1] H NMR SPECTRA OF COMPOUND 4j:

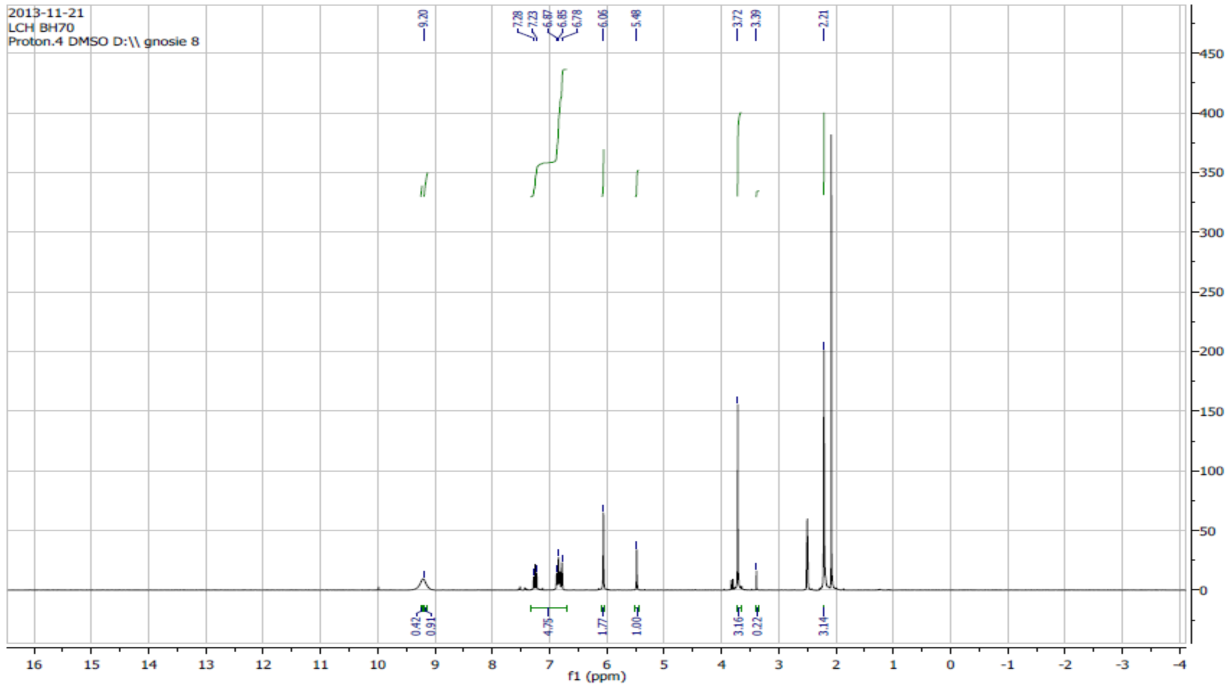

^{13}C NMR SPECTRA OF COMPOUND 4j:

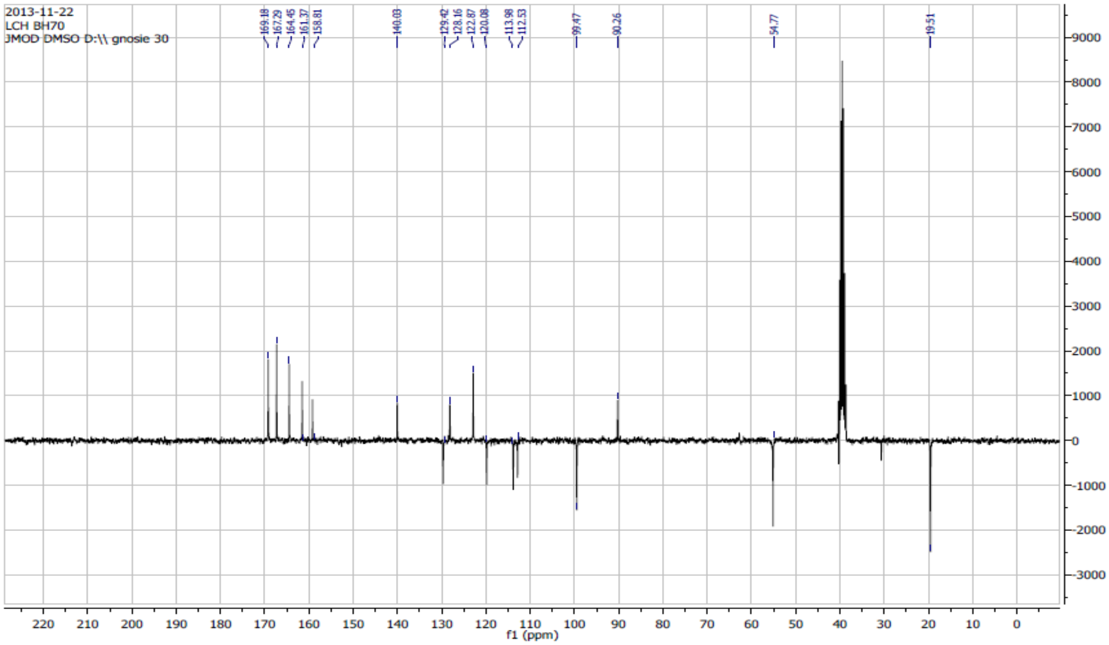

MASS SPECTRA OF COMPOUND 4j:

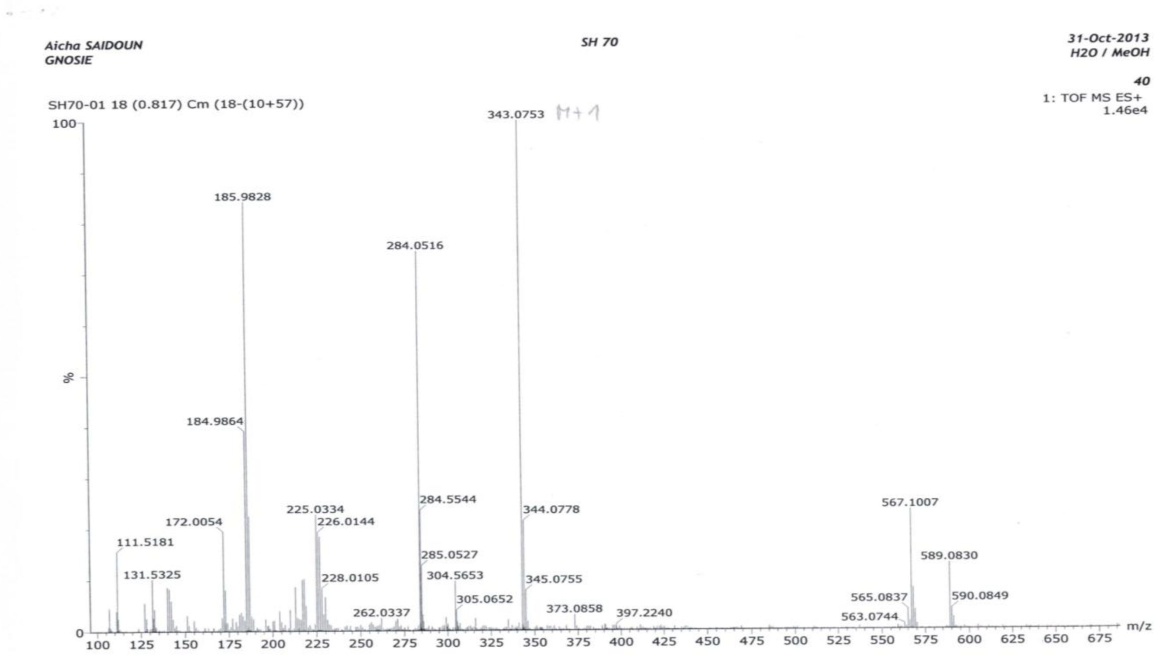

HSQC OF COMPOUND 4f:

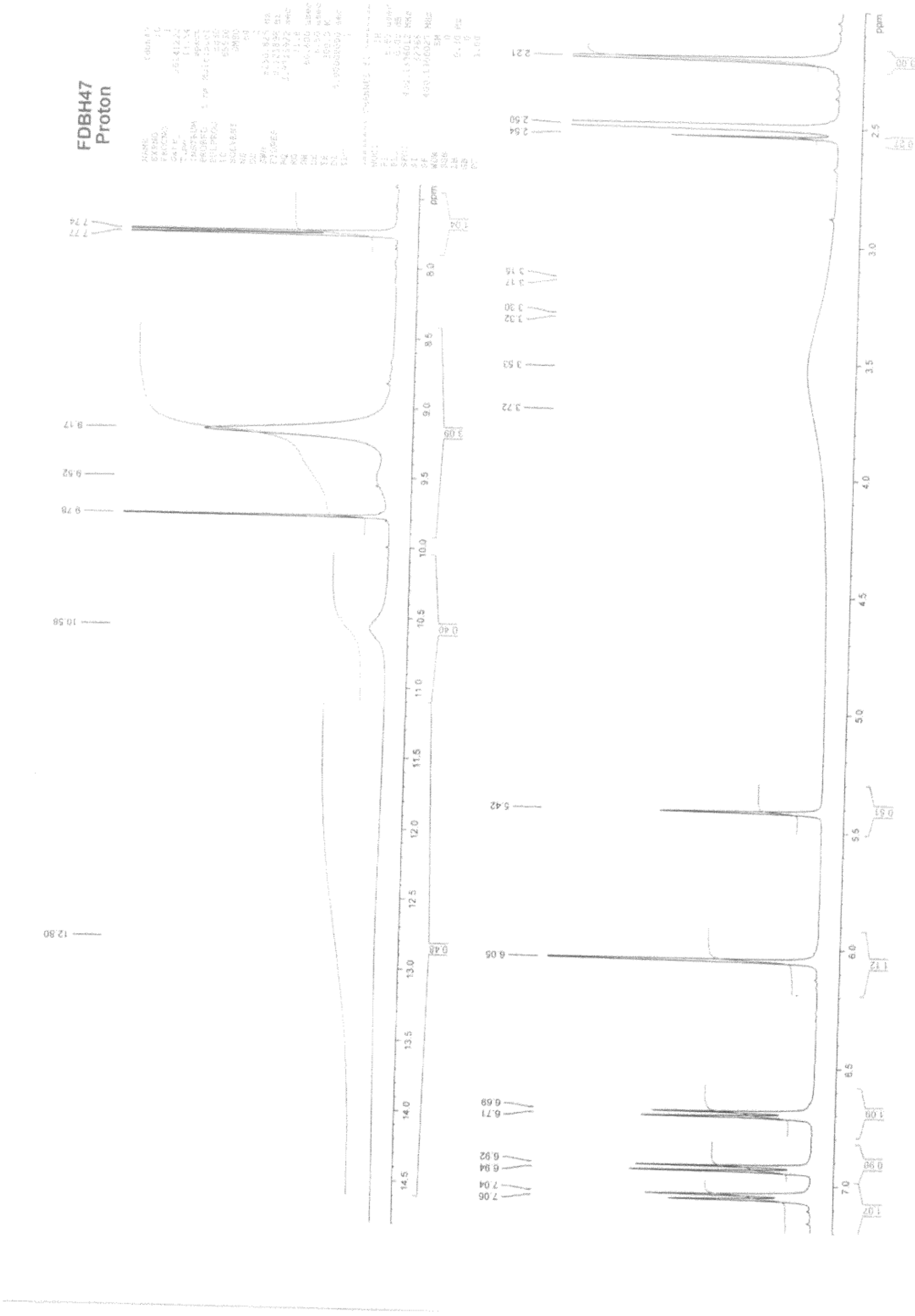

Dec 23, 2014 (9:37:08 AM) fdbh47 10 1 D: ct Page 1/1

Peak	ν(F1) [ppm]	ν(F1) [Hz]	Intensity [abs]
22	12.8043	5123.3846	32699.12
23	10.5772	4232.2551	254122.00
1	9.7833	3914.5919	10182119.12
24	9.5183	3808.5574	199400.38
2	9.1672	3668.0718	2895515.88
3	7.7651	3107.0495	8205069.75
4	7.7438	3098.5267	8345437.75
5	7.0621	2825.7581	6546644.00
6	7.0407	2817.1953	7649246.75
7	6.9421	2777.7425	8107618.75
8	6.9208	2769.2197	7917422.12
9	6.7118	2685.5926	7705741.25
10	6.6905	2677.0698	7264160.12
11	6.0513	2421.3067	14822250.62
12	5.4170	2167.5042	7440566.25
13	3.7154	1486.6430	994943.50
14	3.5308	1412.7790	1280745.12
15	3.3231	1329.6720	943793.25
16	3.3048	1322.3496	907707.38
17	3.1675	1267.4118	574622.38
18	3.1492	1260.0894	543541.50
19	2.5389	1015.8901	7807249.12
20	2.4999	1000.2850	31398129.88
21	2.2108	884.6074	42438570.75

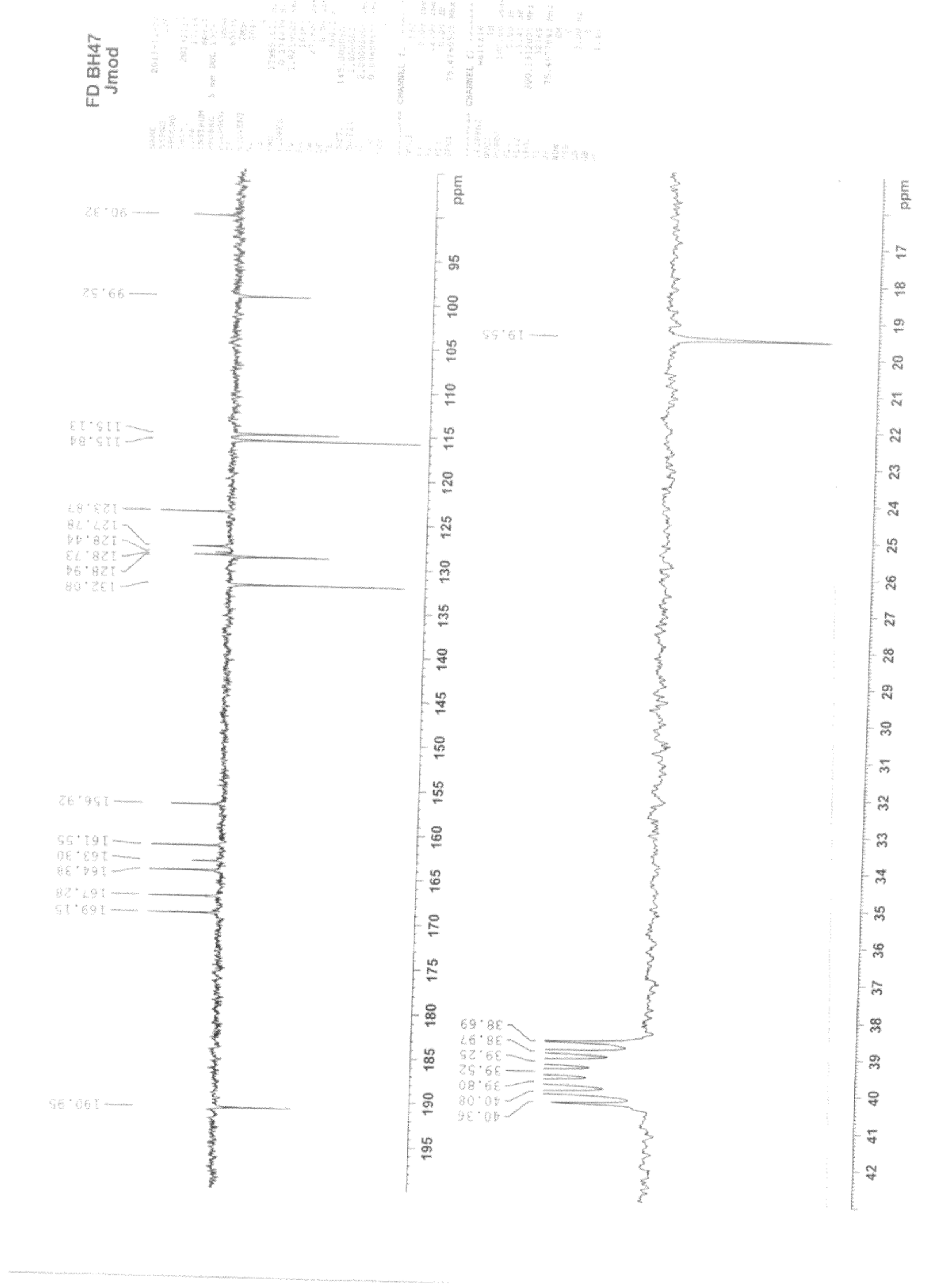
FD BH47
Jmod
90.32
99.52
115.13
115.84
123.87
127.78
128.44
128.73
128.94
132.08
156.92
161.55
163.30
164.38
167.28
169.15
190.85
19.55
38.69
38.97
39.25
39.52
39.80
40.08
40.36
ppm
ppm

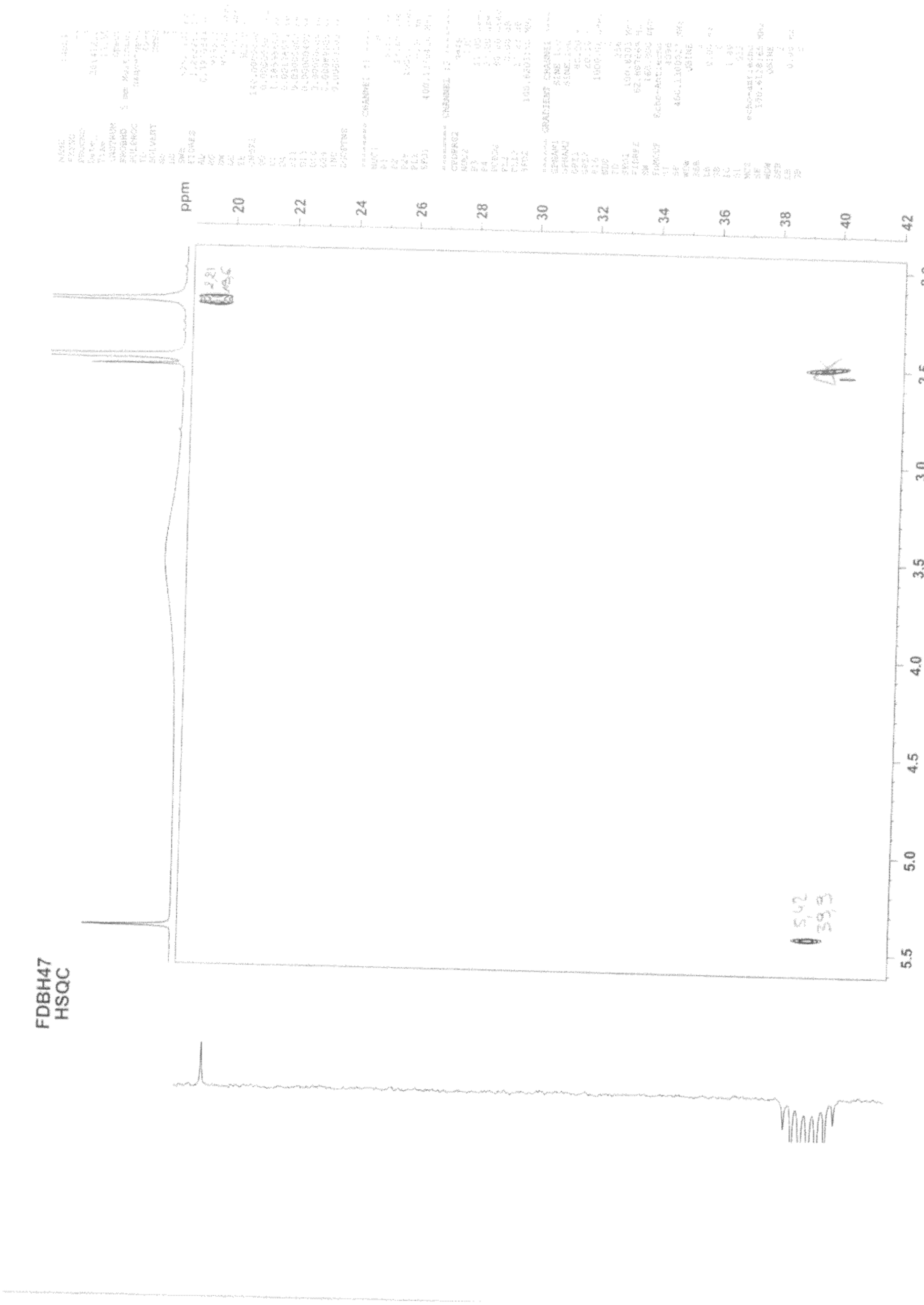
FDBH47
HSQC
ppm
20 22 24 26 28 30 32 34 36 38 40 42
ppm
2.0 2.5 3.0 3.5 4.0 4.5 5.0 5.5

HMBC OF COMPOUND 4f:

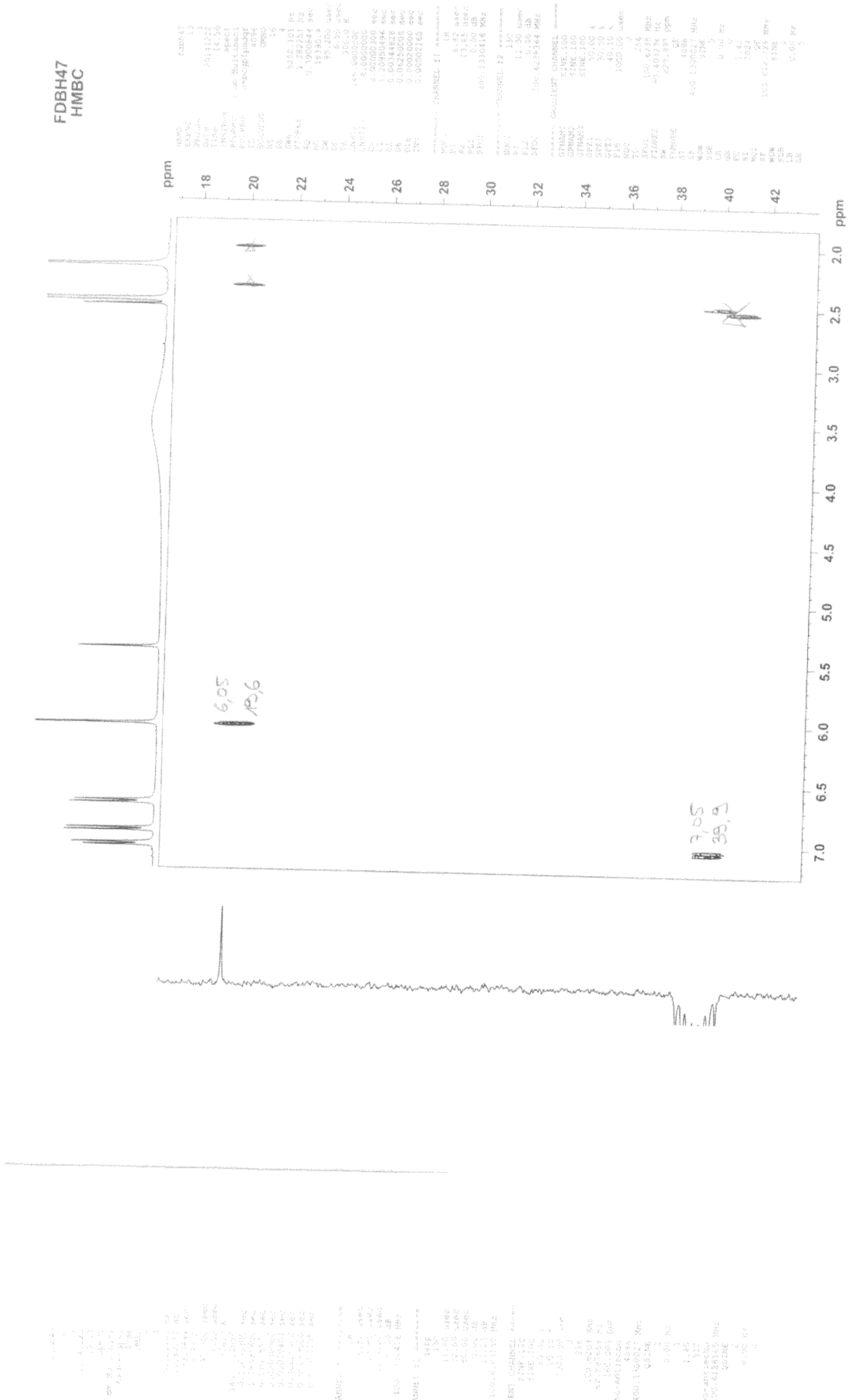

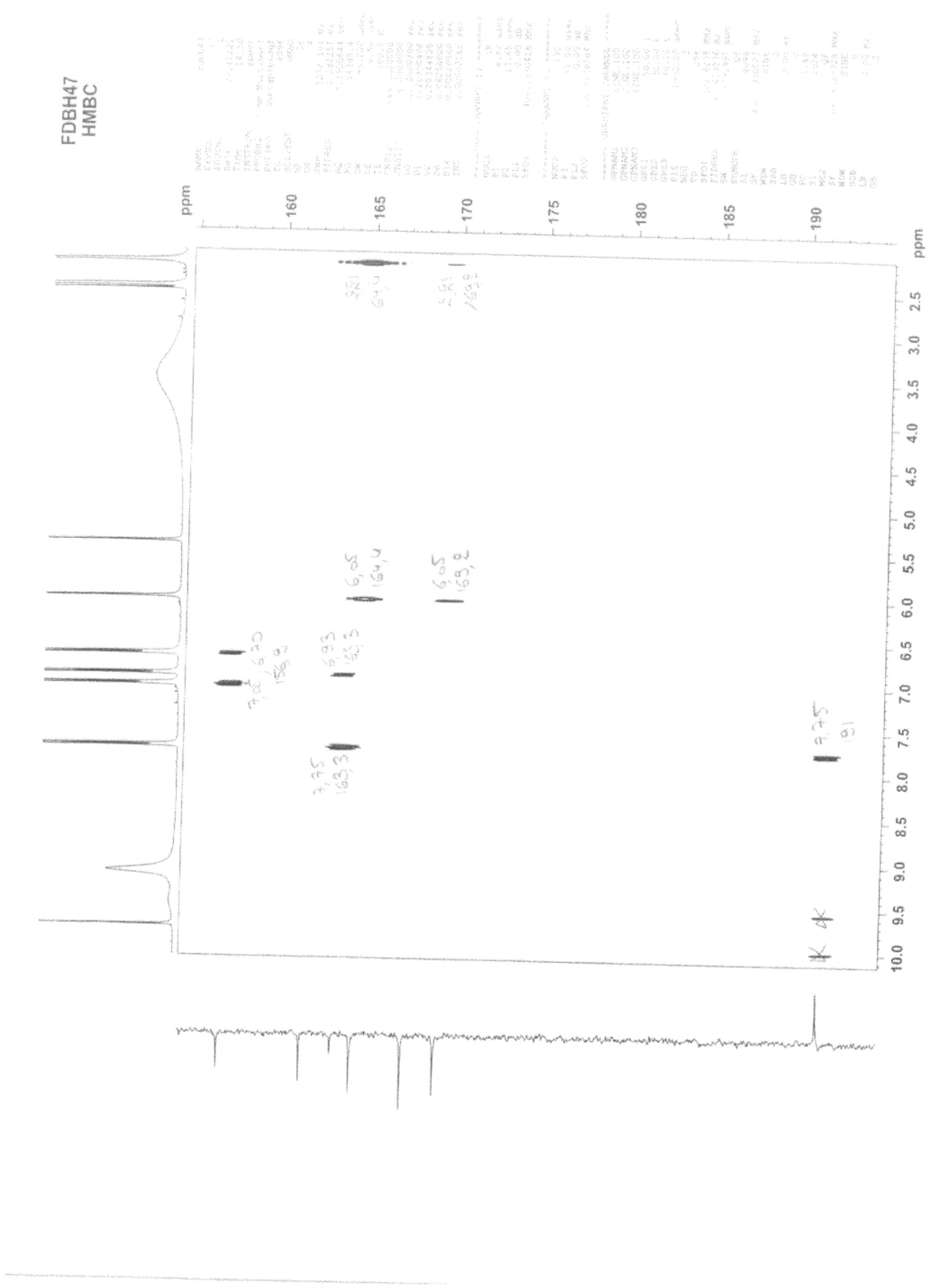
FDBH47
HMBC

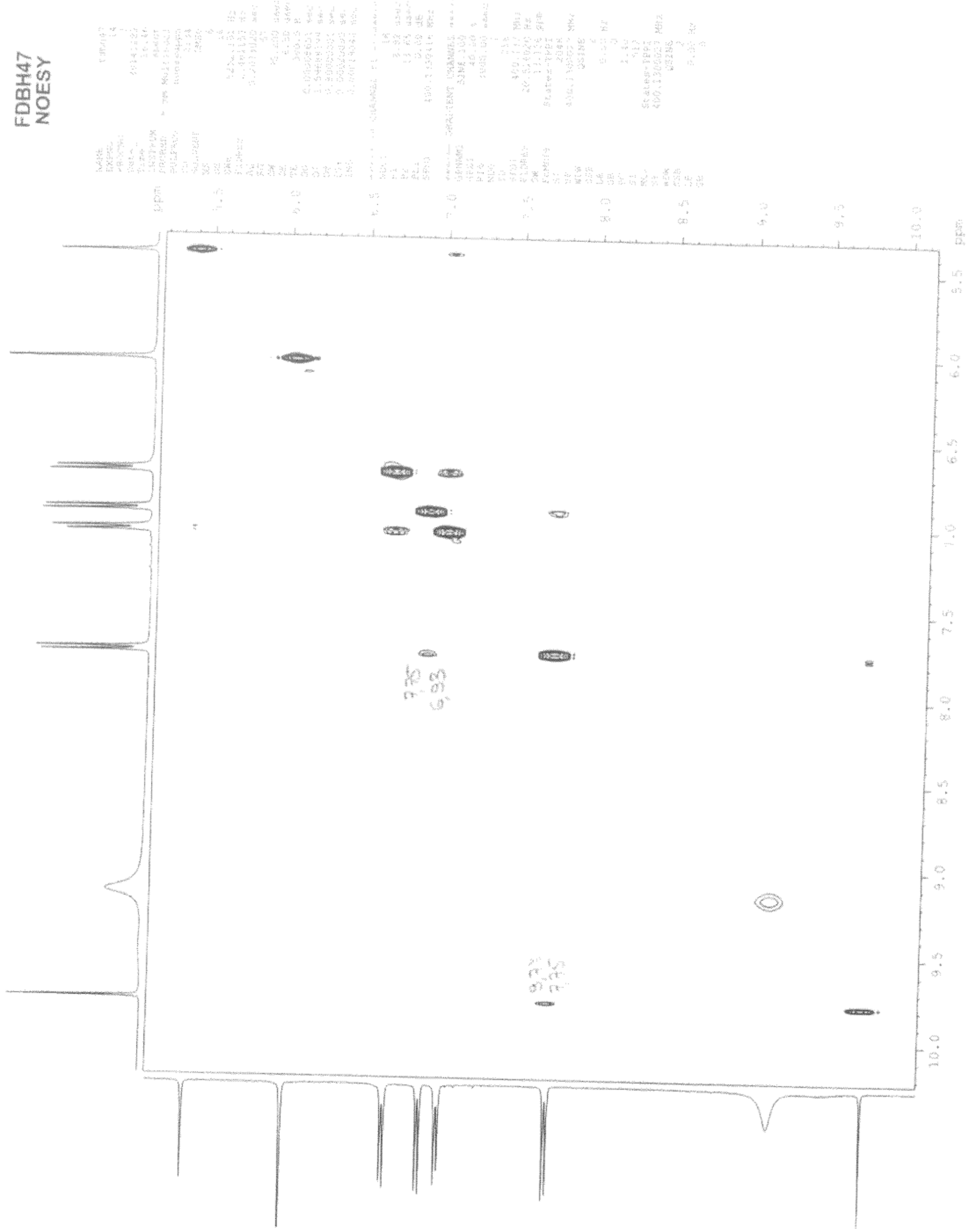
FDBH47
NOESY

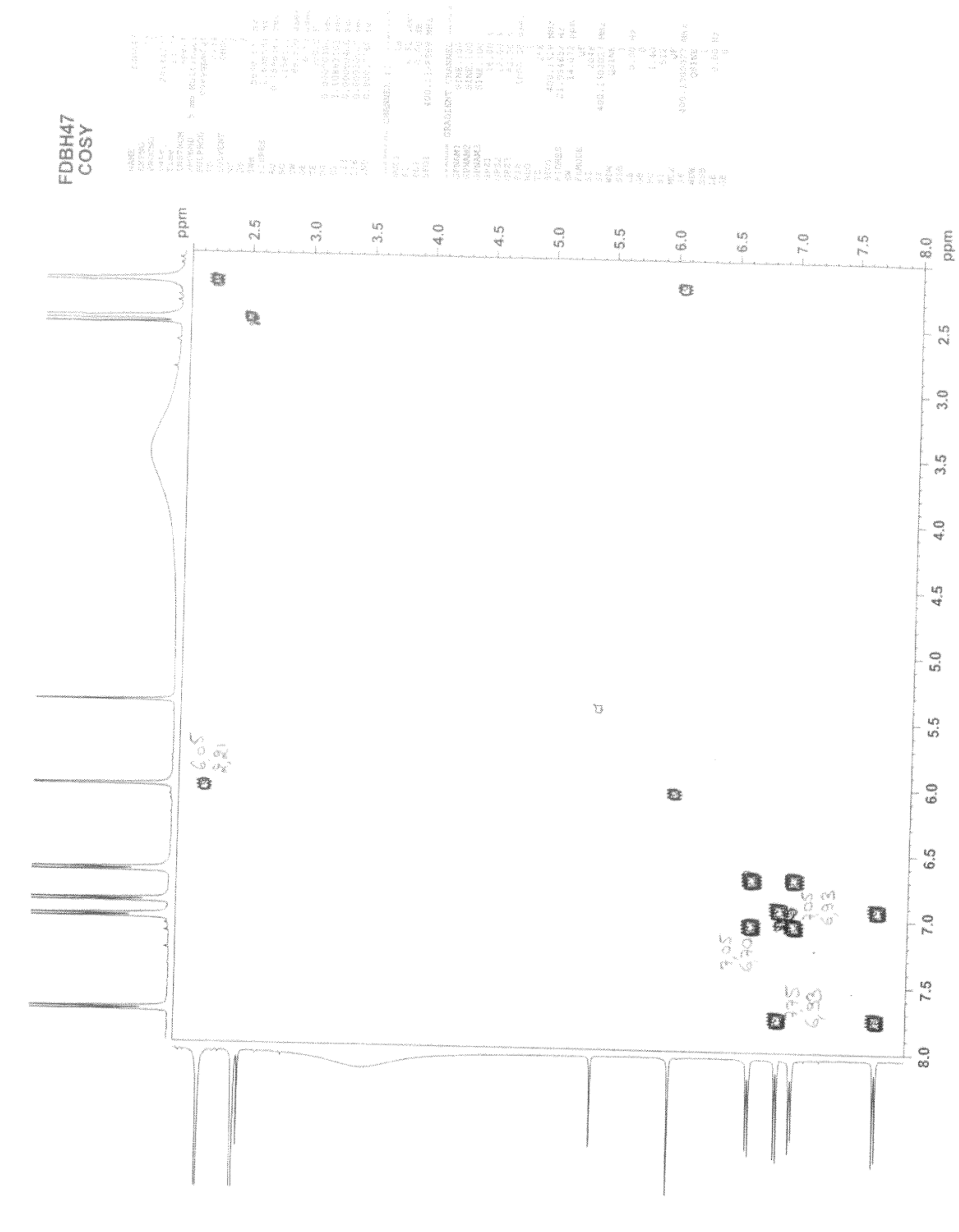
FDBH47
COSY
ppm
ppm
